# On hazard ratio estimators by proportional hazards models in matched-pair cohort studies

**DOI:** 10.1186/s12982-017-0060-8

**Published:** 2017-06-05

**Authors:** Tomohiro Shinozaki, Mohammad Ali Mansournia, Yutaka Matsuyama

**Affiliations:** 1 Department of Biostatistics, School of Public Health, the University of Tokyo, 7-3-1 Hongo, Bunkyo-ku, Tokyo 113-0033 Japan; 2 Department of Epidemiology and Biostatistics, School of Public Health, Tehran University of Medical Sciences, P.O. Box 14155-6446, Tehran, Iran

**Keywords:** Collapsibility, C-statistic, Hazard ratio, Matching, Proportional hazards model

## Abstract

**Background:**

In matched-pair cohort studies with censored events, the hazard ratio (HR) may be of main interest. However, it is lesser known in epidemiologic literature that the partial maximum likelihood estimator of a common HR conditional on matched pairs is written in a simple form, namely, the ratio of the numbers of two pair-types. Moreover, because HR is a noncollapsible measure and its constancy across matched pairs is a restrictive assumption, marginal HR as “average” HR may be targeted more than conditional HR in analysis.

**Methods:**

Based on its simple expression, we provided an alternative interpretation of the common HR estimator as the odds of the matched-pair analog of C-statistic for censored time-to-event data. Through simulations assuming proportional hazards within matched pairs, the influence of various censoring patterns on the marginal and common HR estimators of unstratified and stratified proportional hazards models, respectively, was evaluated. The methods were applied to a real propensity-score matched dataset from the Rotterdam tumor bank of primary breast cancer.

**Results:**

We showed that stratified models unbiasedly estimated a common HR under the proportional hazards within matched pairs. However, the marginal HR estimator with robust variance estimator lacks interpretation as an “average” marginal HR even if censoring is unconditionally independent to event, unless no censoring occurs or no exposure effect is present. Furthermore, the exposure-dependent censoring biased the marginal HR estimator away from both conditional HR and an “average” marginal HR irrespective of whether exposure effect is present. From the matched Rotterdam dataset, we estimated HR for relapse-free survival of absence versus presence of chemotherapy; estimates (95% confidence interval) were 1.47 (1.18–1.83) for common HR and 1.33 (1.13–1.57) for marginal HR.

**Conclusion:**

The simple expression of the common HR estimator would be a useful summary of exposure effect, which is less sensitive to censoring patterns than the marginal HR estimator. The common and the marginal HR estimators, both relying on distinct assumptions and interpretations, are complementary alternatives for each other.

**Electronic supplementary material:**

The online version of this article (doi:10.1186/s12982-017-0060-8) contains supplementary material, which is available to authorized users.

## Background

Matching is a useful sampling method employed in cohort studies, in which the control of confounders is indispensable [1]. The simplest matching design is a 1:1 matched (matched-pair) cohort study, in which each matched pair comprising an exposed and an unexposed member is followed up through the study period. The standard choices of effect measures are common odds ratio (OR) and risk ratio (RR) conditional on matched pairs. As the number of pairs increases, asymptotically unbiased estimate of common OR across matched pairs is the ratio of the number of “discordant” pairs [2, 3]; using the numbers of pairs shown in Table 1, the conditional maximum likelihood estimator (CMLE) of common OR is *B*/*C* [2]. This estimator coincides with the Mantel–Haenszel OR estimator [4] and the unconditional maximum likelihood estimator using multinomial distribution of (*A*, *B*, *C*, *D*) parameterized under common OR [5]. Common RR may be estimated by the Mantel–Haenszel RR estimator, which simplifies to the crude RR (*A* + *B*)/(*A* + *C*) [3, 6], by estimating equations for parameters in conditional multiplicative risk models [7], or by conditional Poisson regression models, which are mimicked by stratified Cox model-fitting with Breslow or Efron-type tie breaking [8, 9].

**Table 1:** Numbers of each pair types in matched-pair cohort data

	Unexposed pair member	
	Event	Nonevent
Exposed pair member		
Event	*A*	*B*
Nonevent	*C*	*D*

One of the concerns in cohort studies is censoring owing to unequal follow-up period or loss to follow-up before the end of the study. In the presence of censoring, common hazard ratio (HR) is a viable alternative. Common HR can be estimated by the Mantel–Haenszel rate ratio [6] or partial maximum likelihood estimators (PMLE) of Cox proportional hazards models stratified on matched pairs [10–12]. However, perhaps because of the ease of Cox model-fitting by modern computer software, it is lesser known that the PMLE of common HR can also be transcribed in a simple formula as in the case of CMLE of common OR [10]. The formula motivates us to focus on the relationship of the PMLE to the matched-pair analog of C-statistics for time-to-event, which has been recently discussed in the literature for evaluating discriminatory ability in prediction [13–15].

This representation would be useful because HR (like OR) is known to be a noncollapsible measure: even under homogeneity of conditional HR across strata and in the absence of confounding, marginal (unadjusted) HR is not necessarily equal to the conditional one [16, 17]. Moreover, the assumption of homogenous (common) HR across strata may be too restrictive. To circumvent interpretational difficulties, marginal HR estimated by unstratified Cox models with robust variance estimator is often of primary interest than common HR [18]. Even when HR is not constant over time, it may be interpreted as the “average” HR of time-varying HR [19]. However, we argue that the uncritical “average” view of marginal HR may have limited value because the estimator depends on censoring distribution that is nuisance to inference for exposure effect on outcome [20, 21].

In this paper, we showed the simple expression of the common HR estimator and its alternative interpretation as the odds of the matched-pair analog of C-statistic for censored time-to-event data. Through simulation studies, assuming proportional hazards within the matched pairs, we evaluated the influence of various censoring patterns on the marginal and common HR estimators of unstratified and stratified proportional hazards models, respectively. For illustration, several estimators were compared in a propensity score-matched dataset of primary breast cancer from the Rotterdam tumor bank.

## Methods

In this section, we provide the simple formula for the common HR estimator under a stratified proportional hazards model in matched-pair cohort studies. The common HR is linked to overall C-index with matched-pair analog to improve its interpretation. By simulation studies under the stratified proportional hazards models, we compare the performance in competing estimators as well as statistical tests used in matched-pair cohort studies in various censoring distributions. Finally, we illustrate the methods in a real dataset.

### Stratified PMLE of common HR in matched-pair cohorts

Consider matched-pair cohort studies comparing time-to-event outcome *T*, in which each pair *k* (*k* = 1,…, *n*) is comprised of an exposed (*e* = 1) and an unexposed member (*e* = 0). Because outcome *T*_*ke*_ of member *e* in pair *k* may be censored by drop-out time *U*_*ke*_, or the end of follow-up *τ*, we observe follow-up time as $X_{ke} = \hbox{min} (T_{ke} ,U_{ke} ,\tau )$. Define *Y*_*ke*_ as an indicator of event (*Y*_*ke*_ = 1 if *X*_*ke*_ = *T*_*ke*_, 0 otherwise). Suppose all risk factors have the same distribution within each pair.

If we are interested in common HR across all matched pairs throughout the follow-up period, an appropriate model is the Cox proportional hazards model stratified on the matched-pair *k*:

1 $$\lambda_{ke} (t) = \lambda_{k0} (t)\exp (\beta \cdot e),$$

where *λ*_*ke*_(*t*) and *β* are a hazard function of *T*_*ke*_ and logarithm of common HR, respectively.

Partial likelihood of (1) is given by the product of the contribution at each event time from each stratum *k*, expressed as follows [10–12]:

2 $$L(\beta ) = \prod\limits_{k = 1}^{n} {L_{k} (\beta )} = \prod\limits_{k = 1}^{n} {\prod\limits_{e = 0,1} {\left\{ {\frac{\exp (\beta \cdot e)}{{\sum\nolimits_{{e^{\prime } \,{\text{in}}\,{\text{pair-}}k\,{\text{risk}}\,{\text{set}}\,{\text{at}}\,X_{ke} }} {\exp (\beta \cdot e^{\prime } )} }}} \right\}^{{Y_{ke} }} } }$$

To express the contribution *L*_*k*_(*β*) from each stratum, we classify each pair observable in matched-pair data into eight types (Table 2). For clarity, the only tie we additionally consider is caused by the end of follow-up, i.e., *X*_*k*1_ = *X*_*k*0_ = *τ* (type 9). Let *n*_1_,…, *n*_9_ denote the number of pairs of types 1–9. Partial likelihood in the presence of other types of ties are shown in “Appendix 1”.

**Table 2:** List of pair types and their contribution to stratified partial likelihood

Type	Number of pairs	Observed data in the pair	Observed time	*Y*_*k*1_	*Y*_*k*0_	*L*_*k*_(*β*)
1	*n*_1_	Exposed gets event first, followed by unexposed event	*X*_*k*1_ < *X*_*k*0_	1	1	$\frac{{{\text{e}}^{\beta } }}{{1 + {\text{e}}^{\beta } }}$
2	*n*_2_	Unexposed gets event first, followed by exposed event	*X*_*k*1_ > *X*_*k*0_	1	1	$\frac{1}{{1 + {\text{e}}^{\beta } }}$
3	*n*_3_	Exposed gets event first, followed by unexposed censored	*X*_*k*1_ < *X*_*k*0_	1	0	$\frac{{{\text{e}}^{\beta } }}{{1 + {\text{e}}^{\beta } }}$
4	*n*_4_	Unexposed gets event first, followed by exposed censored	*X*_*k*1_ > *X*_*k*0_	0	1	$\frac{1}{{1 + {\text{e}}^{\beta } }}$
5	*n*_5_	Exposed is censored first, followed by unexposed event	*X*_*k*1_ < *X*_*k*0_	0	1	1
6	*n*_6_	Unexposed is censored first, followed by exposed event	*X*_*k*1_ > *X*_*k*0_	1	0	1
7	*n*_7_	Exposed is censored first, followed by unexposed censored	*X*_*k*1_ < *X*_*k*0_	0	0	1
8	*n*_8_	Unexposed is censored first, followed by exposed censored	*X*_*k*1_ > *X*_*k*0_	0	0	1
9	*n*_9_	Exposed and unexposed are censored simultaneously	*X*_*k*1_ = *X*_*k*0_ = *τ*	0	0	1

Pairs of types 1 and 3 contribute to partial likelihood by $\frac{\exp (\beta )}{1 + \exp (\beta )}$, pairs 2 and 4 contribute by $\frac{1}{1 + \exp (\beta )}$, and pairs of types 5–9 do not contribute to it. Eventually, the only contributors for the PMLE are those who are in *the pairs in which the pair*-*member with shorter observed time experienced an event*; this is the necessary and sufficient condition for “comparable” pairs in C-statistic for time-to-event, which we revisit later [13–15]. The resulting partial likelihood (2) is

$$\left( {\frac{{{\text{e}}^{\beta } }}{{1 + {\text{e}}^{\beta } }}} \right)^{G} \left( {\frac{1}{{1 + {\text{e}}^{\beta } }}} \right)^{H} ,$$

where *G* = *n*_1_ + *n*_3_ denotes the number of pairs where the exposed member has shorter observed time and experienced an event (types 1 and 3) and *H* = *n*_2_ + *n*_4_ denotes the number of pairs where the unexposed member has shorter observed time and experienced an event (types 2 and 4). Therefore, all information regarding matched-pair data for common HR is concentrated on the number of only two types of pairs.

By maximizing partial likelihood, we can write the PMLE of common HR as *G*/*H*. Substituting *G*/*H* into the observed Fisher information [11], the approximate variance estimator of log(*G*/*H*) can be obtained by 1/*G* + 1/*H*. These are of the same form as the logarithm of the CMLE log(*B*/*C*) and its variance estimator 1/*B* + 1/*C* [2, 3].

### Tests of null association

To test the null hypothesis of common OR in matched-pair data, McNemar’s test is often recommended [22–24]. The test statistic is $\frac{{(B - C)^{2} }}{B + C}$, which is also a function of *B* and *C*. Similarly, by using the definitions of *G* and *H* from above Klein and Moeschberger [12] have developed a stratified log-rank test statistic $\frac{{(G - H)^{2} }}{G + H}$ as a weighted rank statistic. As the number of pairs grows, $\frac{{(G - H)^{2} }}{G + H}$ has an asymptotic Chi-squared distribution with one degree of freedom under *β* = 0. Similar to McNemar’s test that can be considered as the score test of OR = 1 in a conditional logistic model [3], the stratified log-rank test can be considered as the score test for *β* = 0 in a stratified Cox model (1). Note that Wald and score tests for the hypothesis of conditional HR (or OR) = 1 can be shown to be asymptotically equivalent to test statistics for marginal HR (or OR) = 1 [25]. Therefore, tests for both conditional and marginal null hypotheses in different models may be used interchangeably, although OR and HR are both noncollapsible measures.

### Stratified PMLE as overall C-statistic for matched pairs

For binary exposure *E* (1 if exposed, 0 if unexposed) and time-to-event outcome *T*, overall C-index (C-index for time-to-event) is defined as

$${\text{C}}_{\tau } = \hbox{max} \left\{ \begin{aligned} \Pr (E_{i} = 1,\quad E_{j} = 0\left| {T_{i} < T_{j} ,\quad T_{i} < \tau } \right.) \hfill \\ \Pr (E_{i} = 0,\quad E_{j} = 1\left| {T_{i} < T_{j} ,\quad T_{i} < \tau } \right.) \hfill \\ \end{aligned} \right\}$$

where *τ* is the time of the end of follow-up or an arbitrary time interval set by analysts [13, 14]. Assuming the absence of censoring except at the end of follow-up, Pencina and D’Agostino proposed to estimate C_*τ*_ by restricting all possible pairs in the sample to “comparable” pairs, in which the member with a shorter observed time experienced an event, i.e., “$X_{i} < X_{j} ,Y_{i} = 1,X_{i} < \tau$” [14].

We can consider the matched-pair analog of C_*τ*_:

$${\text{C}}_{{\tau ,{\text{pair}}}} = \hbox{max} \left\{ \begin{aligned} \Pr (e_{1} = 1,\quad e_{2} = 0\,{\text{in}}\,{\text{pair}}\,k\left| {T_{{ke_{1} }} < T_{{ke_{2} }} ,\quad T_{{ke_{1} }} < \tau } \right.) \hfill \\ \Pr (e_{1} = 0,\quad e_{2} = 1\,{\text{in}}\,{\text{pair}}\,k\left| {T_{{ke_{1} }} < T_{{ke_{2} }} ,\quad T_{{ke_{1} }} < \tau } \right.) \hfill \\ \end{aligned} \right\}$$

where (*e*_1_, *e*_2_) is either (0, 1) or (1, 0), and sampling is made for matched pairs *k* = 1,…, *n*. Let *S*_*ke*_(*t*) and *f*_*ke*_(*t*) denote survival and density functions of *T*_*ke*_, respectively. Given the matched-pair design considered here, $\Pr (e_{1} = 1,\quad e_{2} = 0\,{\text{in}}\,{\text{pair}}\,k\left| {T_{{ke_{1} }} < T_{{ke_{2} }} ,\quad T_{{ke_{1} }} < \tau } \right.)$ is expressed as $\Pr (T_{k1} < T_{k0} \left| {T_{k1} < \tau \,{\text{or}}\,T_{k0} < \tau } \right.) = \frac{{\Pr (T_{k1} < T_{k0} ,T_{k1} < \tau )}}{{1 - S_{k0} (\tau )S_{k1} (\tau )}}$. Taking the odds of this probability yields the following under the stratified Cox model (1):

$$\begin{aligned} \frac{{\Pr (T_{k1} < T_{k0} ,\quad T_{k1} < \tau )}}{{\Pr (T_{k1} > T_{k0} ,\quad T_{k0} < \tau )}} & = \frac{{\int_{0}^{\tau } {f_{k1} (t)S_{k0} (t)dt} }}{{\int_{0}^{\tau } {f_{k0} (t)S_{k1} (t)dt} }} \\ & = \frac{{\int_{0}^{\tau } {\{ \lambda_{k0} (t){\text{e}}^{\beta } S_{k1} (t)\} S_{k0} (t)dt} }}{{\int_{0}^{\tau } {\{ \lambda_{k0} (t)S_{k0} (t)\} S_{k1} (t)dt} }} \\ & = {\text{e}}^{\beta } . \\ \end{aligned}$$

Therefore, the common HR exp(*β*) equals C_*τ*,pair_/(1 − C_*τ*,pair_) if common HR > 1, and (1 − C_*τ*,pair_)/C_*τ*,pair_ if common HR < 1. In fact, this relationship has been derived by Holt and Prentice over 40 years ago for *τ* = ∞, in which case ${\text{C}}_{{\tau , {\text{pair}}}} = \Pr (T_{k1} < T_{k0} )$ if common HR > 1 and ${\text{C}}_{{\tau , {\text{pair}}}} = \Pr (T_{k1} > T_{k0} )$ otherwise [10].

Due to censoring by *U*_*ke*_, event times *T*_*k*1_ and *T*_*k*0_ are not always observable. As shown in the “Appendix 2”, *G*/(*G* + *H*) converges in probability (as pair number *n* grows) to

3 $$\begin{aligned} \Pr \{ e_{1} = 1,e_{2} = 0\,\quad{\text{in}}\,{\text{pair}}\,k\left| {T_{{ke_{1} }} < T_{{ke_{2} }} ,T_{{ke_{1} }} < \hbox{min} (U_{{ke_{1} }} ,U_{{ke_{2} }} ),T_{{ke_{1} }} < \tau } \right.\} \hfill \\ = \Pr \{ T_{k1} < T_{k0} \left| {T_{k1} < \hbox{min} (U_{k1} ,U_{k0} ,\tau )\,{\text{or}}\,T_{k0} < \hbox{min} (U_{k1} ,U_{k0} ,\tau )} \right.\} \hfill \\ \end{aligned}$$

Note that (3) is not equal to $\Pr (T_{k1} < T_{k0} \left| {T_{k1} < \tau {\text{ or }}T_{k0} < \tau } \right.)$ in general. Thus, C_*τ*,pair_ cannot be apparently calculated by the observed data if censoring before *τ* occurs. “Appendix 2” shows, however, that under the model (1), the odds of (3) equal exp(*β*) even if *T*_*ke*_ is censored by *U*_*ke*_ that is independent to *T*_*ke*_ conditional on matched pairs and exposure. Thus, we can estimate C_*τ*,pair_ as well as *β* based on only “comparable” matched pairs introduced by design even if censoring depends on both matched pairs and exposure.

### Simulation studies

To examine the performance of the stratified PMLE under the assumption (1) compared to competitive PMLEs used in matched-pair cohort studies, we simulated 2000 cohorts with size 2*n* = 100, 500 (*n* = 50, 250 exposed–unexposed pairs). SAS code for generating data will be provided in the Additional file 1.

We simulated each pair’s “effect” *γ*_*k*_ as a standard normal variate, assuming that matching eliminates all confounding, though the assumption is at best expected to approximately hold in practice. Time-to-event was then generated from the random-intercept (frailty) model [11, 12] $\lambda_{ke} (t) = \lambda_{0} \exp (\gamma_{k} )\exp (\beta \cdot e)$ with *λ*_0_ = 1 and common log-HR *β* = log(2.0), log(1.0), and log(0.5). The time was censored by exponential variate according to the following censorship patterns:

1. Independent censoring with the rate parameter of 1, 2, and 4, or
2. Conditionally independent censoring given strata and exposure, where the rate parameter equals *γ*_*k*_ + *αe*, *α* = log(0.25), log(1.0), and log(4.0).

We also employed Weibull time-to-event variables in additional scenarios to emulate the situations in which (1) baseline hazards increase or decrease instead of the time-constant hazard *λ*_0_, or (2) the shape parameter varied between the strata while keeping stratum-specific HR fixed as a constant across strata. As the results from these additional scenarios were similar to those from the above exponential-normal frailty model, the parameter settings and the results are provided in the Additional file 1.

We fitted pair-stratified Cox models, unstratified Cox models with or without robust sandwich variance estimator [26], as well as true frailty Cox models as a reference. Note that stratified and frailty models assume that the conditional parameter is constant across matched pairs, while unstratified models only model a marginal parameter and do not assume such constancy across pairs.

With the frailty Cox models used in the data generation, the marginal distributions of time do not follow proportional hazards except for the positive-stable distributed frailty [12]. Thus, the unstratified Cox model is known to be misspecified. One way around this problem is to define the model parameters as the asymptotic means of the maximum-likelihood estimators that are free from censoring, which is always well-defined and interpretable (even if the models are not correct) [20, 27, 28]. Therefore, the targeted marginal HR in this study is defined as a mean of the estimate of unstratified Cox models calculated in a large (*n* = 5,000,000) dataset where no member is censored.

The performance of the above estimators was evaluated by mean bias (the average of 2000 log-HR estimates—true log HR), empirical standard error (standard deviation of 2000 estimates), mean estimated standard error, root mean squared error (RMSE; the square root of the sum of the squared bias and the empirical variance of the estimator), and coverage proportion of 95% confidence intervals. The empirical power (or type I error when HR = 1) tested by Wald statistics (log-HR estimates divided by their estimated standard errors) of the above was also compared, accompanied by a stratified log-rank test statistic. We used PHREG procedure in SAS version 9.4 (Cary, NC).

### Application: propensity-matched cohort data from the Rotterdam tumor bank

To illustrate the methods in a real dataset, we used the records from the Rotterdam tumor bank, which includes follow-up data on 2982 women with primary breast cancer. The dataset is available in the R package AF developed by Dahlqwist and Sjölander [29] and the details of the dataset have been described elsewhere [30]. The outcome *T* is relapse-free survival, which is defined as time to developing relapse of breast cancer or death from any cause before the end of the follow-up period. Women remained in the dataset until they experienced relapse or death, were lost to follow-up or were at the end of the follow-up period, whichever came first. The exposure of interest is the absence of chemotherapy (1 if treated without chemotherapy, *n* = 2402; 0 if treated with chemotherapy, *n* = 580).

One notable feature in this dataset is that the amount of confounding is very strong—adjusting for the possible confounders reverses the sign of the association [30]. Thus, we turned the dataset into a matched cohort based on propensity score (the conditional probability of exposure given possible confounders). Propensity score is conditional on the following prognostic variables: age at surgery (years), menopausal status (0 if premenopausal, 1 if postmenopausal), tumor size (≤20 mm, >20–50 mm, and >50 mm), tumor grade (2 or 3), progesterone receptors, (fmol/l), oestrogen receptors (fmol/l), and the number of positive lymph nodes [ranging between 0 and 34; transformed into exp(–0.12 * the number of nodes)]. We estimated the propensity score for each woman by fitting a logistic model, and then matched women on the estimated propensity scores by caliper-based pair-matching algorithm without replacement (allowable caliper width was 20% of the standard deviation of estimated propensity scores in a chemotherapy group). The resulting matched cohort is comprised of *n* = 446 exposed–unexposed pairs. The SAS code for forming the propensity-matched cohort from the Rotterdam dataset is provided in the Additional file 1.

## Results

### Simulation results

Table 3 shows the results in independently censored data (similar results were obtained for *n* = 50, provided in online supplementary material). The marginal HR defined in the unstratified models is towards null from conditional HR, similar to the well-known result that the marginal OR is closer to null than common stratum-specific OR [16]. In fact, marginal HR always lies between the conditional HR and 1 under the exponential survival model [17]. Null exposure effect in conditional HR implies that marginal HR is also null. In this case, no estimator has a bias. Coverage of confidence intervals maintains almost nominal level except for the unstratified model without robust variance that overestimates the true variability.

**Table 3:** Simulated estimates with independent censoring distribution, varying censoring rate (2000 repetitions, *n* = 250)

Method	Censoring rate	MCSE	MESE	Log conditional-HR			Log marginal-HR		
				Bias	95% CP (%)	RMSE	Bias	95% CP (%)	RMSE
	Log conditional-HR = log(2) = 0.693; Log marginal-HR = 0.437								
Frailty Cox model	1	0.13	0.13	−0.03	93.50	0.14	0.23	58.80	0.27
Stratified Cox model		0.18	0.18	0.00	95.20	0.18	0.26	72.15	0.32
Unstratified Cox model without sandwich variance		0.10	0.12	−0.19	66.15	0.22	0.07	95.35	0.12
Unstratified Cox model with sandwich variance		0.10	0.10	−0.19	51.80	0.22	0.07	90.75	0.12
Frailty Cox model	2	0.15	0.15	−0.03	94.49	0.15	0.22	68.69	0.27
Stratified Cox model		0.21	0.20	0.01	95.45	0.21	0.26	76.85	0.34
Unstratified Cox model without sandwich variance		0.12	0.14	−0.16	80.70	0.20	0.09	93.25	0.15
Unstratified Cox model with sandwich variance		0.12	0.12	−0.16	70.90	0.20	0.09	88.40	0.15
Frailty Cox model	4	0.17	0.18	−0.04	95.19	0.18	0.22	78.81	0.28
Stratified Cox model		0.25	0.25	0.01	94.85	0.25	0.26	83.40	0.37
Unstratified Cox model without sandwich variance		0.15	0.17	−0.13	89.55	0.20	0.12	91.95	0.19
Unstratified Cox model with sandwich variance		0.15	0.15	−0.13	84.20	0.20	0.12	87.35	0.19
	Log conditional-HR = log(1) = 0; Log marginal-HR = 0								
Frailty Cox model	1	0.14	0.14	0.00	95.55	0.14	0.00	95.55	0.14
Stratified Cox model		0.18	0.18	0.00	95.75	0.18	0.00	95.75	0.18
Unstratified Cox model without sandwich variance		0.11	0.13	0.00	97.70	0.11	0.00	97.70	0.11
Unstratified Cox model with sandwich variance		0.11	0.11	0.00	94.55	0.11	0.00	94.55	0.11
Frailty Cox model	2	0.15	0.16	0.00	95.34	0.15	0.00	95.34	0.15
Stratified Cox model		0.21	0.21	0.00	95.30	0.21	0.00	95.30	0.21
Unstratified Cox model without sandwich variance		0.13	0.15	0.00	97.45	0.13	0.00	97.45	0.13
Unstratified Cox model with sandwich variance		0.13	0.13	0.00	94.40	0.13	0.00	94.40	0.13
Frailty Cox model	4	0.19	0.19	0.00	95.78	0.19	0.00	95.78	0.19
Stratified Cox model		0.27	0.26	0.00	95.65	0.27	0.00	95.65	0.27
Unstratified Cox model without sandwich variance		0.17	0.18	0.00	97.10	0.17	0.00	97.10	0.17
Unstratified Cox model with sandwich variance		0.17	0.17	0.00	95.05	0.17	0.00	95.05	0.17
	Log conditional-HR = log(0.5) = –0.693; Log marginal-HR = –0.438								
Frailty Cox model	1	0.15	0.15	0.04	94.19	0.15	−0.22	69.19	0.27
Stratified Cox model		0.20	0.20	0.00	95.30	0.20	−0.26	76.65	0.33
Unstratified Cox model without sandwich variance		0.12	0.14	0.17	80.55	0.21	−0.09	94.20	0.15
Unstratified Cox model with sandwich variance		0.12	0.12	0.17	70.35	0.21	−0.09	89.25	0.15
Frailty Cox model	2	0.17	0.18	0.04	94.13	0.18	−0.21	79.49	0.28
Stratified Cox model		0.25	0.25	−0.01	95.10	0.25	−0.26	83.30	0.36
Unstratified Cox model without sandwich variance		0.15	0.17	0.14	89.95	0.20	−0.12	92.25	0.19
Unstratified Cox model with sandwich variance		0.15	0.15	0.14	84.90	0.20	−0.12	88.50	0.19
Frailty Cox model	4	0.22	0.22	0.03	94.97	0.22	−0.22	84.80	0.31
Stratified Cox model		0.31	0.31	−0.01	95.45	0.31	−0.27	88.55	0.41
Unstratified Cox model without sandwich variance		0.19	0.21	0.10	93.35	0.22	−0.16	91.90	0.25
Unstratified Cox model with sandwich variance		0.19	0.20	0.10	91.05	0.22	−0.16	88.40	0.25

MCSE, empirical (Monte Carlo) standard error; MESE, mean estimated standard error; 95% CP, coverage proportion of 95% confidence interval; RMS,E root mean square error

For non-null HR (*β* ≠ 0), PMLE for unstratified models have “bias” from conditional HR that partly reflects the noncollapsible property of HR [17] and the dependency on censoring distribution. The latter also impedes its interpretation as “average” marginal HR that is independent of censoring. Frailty to disease structurally changes hazard among the remaining risk-set over the follow-up period [19, 31]: under our simulation model, HR constancy during the follow-up period only holds conditionally on frailty but does not hold marginally with non-null exposure effect. Estimates for unstratified models are indeed valid as marginal effect-measures in pair-matched data if there are no other covariates that need to be controlled *and* if no censoring occurs [20, 32]. If no observation is censored, estimates from unstratified models are unbiased for the marginal HR parameter (data not shown). As censoring increases, the bias in unstratified PMLE from the marginal HR parameter becomes larger and the coverage probability decreases.

Table 4 shows the results for censorship dependent on matched pair and exposure. The pair effect on censoring alone (from the rows “Censoring rate ratio = 1”) does not invalidate any estimate for null exposure effect but biases unstratified PMLE from both conditional and marginal HRs under non-null exposure effect, as expected from Table 3. Exposure effect on censoring also affects the distribution of unstratified PMLE for both null and non-null exposure effects. This censoring mechanism also makes bias in PMLE for frailty Cox models despite that it models true hazard. Only stratified PMLE, *G*/*H*, has no bias in this censoring pattern, which is guaranteed with the assumption (1), as shown in “Appendix 2”.

**Table 4:** Simulated estimates with conditionally independent censoring given matched-pair and exposure, varying censoring rate ratio by exposure (2000 Repetitions, *n* = 250)

Method	Censoring rate ratio by exposure	MCSE	MESE	Log conditional-HR			Log marginal-HR		
				Bias	95% CP (%)	RMSE	Bias	95% CP (%)	RMSE
	Log conditional-HR = log(2) = 0.693; Log marginal-HR = 0.437								
Frailty Cox model	0.25	0.12	0.12	0.09	88.60	0.16	0.35	18.15	0.37
Stratified Cox model		0.16	0.16	0.00	94.95	0.16	0.26	65.90	0.30
Unstratified Cox model without sandwich variance		0.10	0.11	−0.04	96.50	0.10	0.21	52.90	0.23
Unstratified Cox model with sandwich variance		0.10	0.09	−0.04	91.55	0.10	0.21	37.75	0.23
Frailty Cox model	1	0.13	0.13	−0.02	94.20	0.13	0.24	54.85	0.27
Stratified Cox model		0.18	0.17	0.00	94.85	0.18	0.26	70.50	0.31
Unstratified Cox model without sandwich variance		0.10	0.12	−0.15	78.80	0.18	0.11	89.05	0.15
Unstratified Cox model with sandwich variance		0.10	0.10	−0.15	68.55	0.18	0.11	82.65	0.15
Frailty Cox model	4	0.16	0.16	−0.27	58.60	0.31	−0.01	95.17	0.16
Stratified Cox model		0.22	0.22	0.01	95.35	0.22	0.26	80.00	0.34
Unstratified Cox model without sandwich variance		0.14	0.15	−0.39	22.85	0.41	−0.13	87.40	0.19
Unstratified Cox model with sandwich variance		0.14	0.13	−0.39	17.95	0.41	−0.13	83.60	0.19
	Log conditional-HR = log(1) = 0; Log marginal-HR = 0								
Frailty Cox model	0.25	0.13	0.12	0.14	79.60	0.19	0.14	79.60	0.19
Stratified Cox model		0.16	0.16	0.00	95.40	0.16	0.00	95.40	0.16
Unstratified Cox model without sandwich variance		0.10	0.12	0.17	71.00	0.20	0.17	71.00	0.20
Unstratified Cox model with sandwich variance		0.10	0.10	0.17	59.65	0.20	0.17	59.65	0.20
Frailty Cox model	1	0.13	0.14	0.00	95.34	0.13	0.00	95.34	0.13
Stratified Cox model		0.18	0.18	0.00	95.50	0.18	0.00	95.50	0.18
Unstratified Cox model without sandwich variance		0.11	0.13	0.00	97.75	0.11	0.00	97.75	0.11
Unstratified Cox model with sandwich variance		0.11	0.11	0.00	95.20	0.11	0.00	95.20	0.11
Frailty Cox model	4	0.18	0.18	−0.29	63.82	0.34	−0.29	63.82	0.34
Stratified Cox model		0.24	0.24	0.00	95.80	0.24	0.00	95.80	0.24
Unstratified Cox model without sandwich variance		0.16	0.17	−0.33	52.20	0.36	−0.33	52.20	0.36
Unstratified Cox model with sandwich variance		0.16	0.16	−0.33	47.00	0.36	−0.33	47.00	0.36
	Log conditional-HR = log(0.5) = –0.693; Log marginal-HR = –0.438								
Frailty Cox model	0.25	0.14	0.13	0.19	68.73	0.24	−0.06	91.75	0.15
Stratified Cox model		0.18	0.18	0.00	95.50	0.18	−0.26	72.70	0.31
Unstratified Cox model without sandwich variance		0.11	0.12	0.35	15.30	0.37	0.10	90.70	0.14
Unstratified Cox model with sandwich variance		0.11	0.11	0.35	9.60	0.37	0.10	84.75	0.14
Frailty Cox model	1	0.15	0.15	0.02	94.66	0.15	−0.23	65.46	0.28
Stratified Cox model		0.21	0.21	0.00	95.10	0.21	−0.26	77.85	0.33
Unstratified Cox model without sandwich variance		0.13	0.14	0.11	90.35	0.17	−0.15	85.50	0.20
Unstratified Cox model with sandwich variance		0.13	0.13	0.11	85.85	0.17	−0.15	79.80	0.20
Frailty Cox model	4	0.22	0.22	−0.29	76.15	0.36	−0.55	27.32	0.59
Stratified Cox model		0.28	0.28	−0.01	95.35	0.28	−0.26	86.80	0.39
Unstratified Cox model without sandwich variance		0.21	0.21	−0.29	77.05	0.35	−0.54	25.40	0.58
Unstratified Cox model with sandwich variance		0.21	0.21	−0.29	74.15	0.35	−0.54	22.20	0.58

MCSE, empirical (Monte Carlo) standard error; MESE, mean estimated standard error; 95%CP, coverage proportion of 95% confidence interval; RMSE, root mean square error

The shortcomings of stratified and frailty PMLEs are their variability. Even if conditional HR is of primary interest, their RMSE can be greater than that from unstratified models. However, in the moderate sized samples (e.g., *n* > 250), the variability around conditional HR by stratified and frailty PMLEs can be outweighed by the bias in unstratified models: the “bias” from conditional and marginal HRs. Frailty models also failed to converge a few times in 2000 repetitions.

### Matched analysis of the Rotterdam cohort

**Fig. 1:**
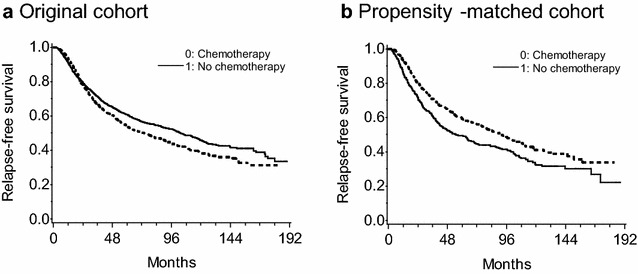
The Kaplan–Meier estimates of relapse-free survival from **a** the original and **b** the propensity-matched cohorts from the Rotterdam tumor bank dataset

The Kaplan–Meier estimates of relapse-free survival from the original (2982 women) and the propensity-matched (2*n* = 892 women) Rotterdam cohorts were depicted in Fig. 1. While the unadjusted curves in the original cohorts favored the absence of chemotherapy, the propensity-matched curves adjusting possible confounders reversed the association of exposure and outcome.

**Fig. 2:**
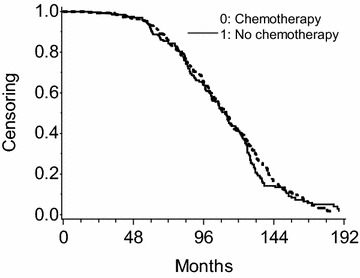
The Kaplan–Meier estimates of censoring distribution from the propensity-matched Rotterdam dataset

Censoring before the end of the follow-up period was not negligible in the matched cohort, but censoring distribution is similar across exposure groups (Fig. 2): the situation would resemble the simulation pattern 1 (independent censoring). Among 446 pairs in the matched cohort, the number of pairs where the exposed member has shorter observed time and experienced an event (*G*) was 198 and the number of pairs where the unexposed member has shorter observed time and experienced an event (*H*) was 135. The PMLE of common HR is *G*/*H* = 1.47 with 95% confidence limits exp(log(1.47) ± 1.96 × √(1/198 + 1/135)) = 1.18 and 1.83; these estimates coincide with the result from the stratified Cox model fitting program in SAS/PHREG procedure. On the contrary, marginal HR estimated from the unstratified Cox model with robust sandwich variance estimator in the same matched cohort is 1.33 (95% CI 1.13–1.57). As seen from simulation, the common HR estimate was further from null than marginal HR estimate in this dataset.

These results were compared to other marginal or conditional HR estimates adjusting for the seven prognostic variables: Table 5 shows the estimates from inverse-probability weighted Cox model with robust variance estimator, multivariable-adjusted Cox model, and multivariable-adjusted Cox model with inverse-probability weighting and robust variance estimator. Although the target populations were not the same between propensity-matched and inverse-probability weighted analyses [18, 33], the PMLE of stratified and unstratified Cox models from the matched cohort approximate the conditional and marginal estimates from the original cohort, respectively.

**Table 5:** Hazard ratio estimates from the Rotterdam tumor bank dataset

Model	Analysis set	Target HR	HR estimate	95% CI	
Stratified Cox model^a^	Matched cohort	Conditional^e^	1.47	1.18	1.83
Unstratified Cox model^b^	Matched cohort	Marginal^e^	1.33	1.13	1.57
IPW Cox model^c^	Original cohort	Marginal^f^	1.32	1.07	1.62
Multivariable Cox model^d^	Original cohort	Conditional^f^	1.48	1.27	1.71
IPW multivariable Cox model^c,d^	Original cohort	Conditional^f^	1.58	1.28	1.96
Unadjusted Cox model	Original cohort	Biased	0.84	0.75	0.95

*CI* confidence interval, *IPW* inverse-probability weighted, *HR* hazard ratio

^a^Stratified on matched pairs

^b^Using a robust variance estimator aggregating residuals within pairs

^c^Using a robust variance estimator aggregating residuals within an individual woman

^d^Adjusted for age at surgery, menopausal status, tumor size, tumor grade, progesterone receptors, oestrogen receptors, and exp(–0.12 * the number of positive lymph nodes). Age and the transformed number of nodes were included by linear and quadratic terms

^e^Target population is the matched part of unexposed population (treated with chemotherapy)

^f^Target population is total (unexposed and exposed) population

## Discussion

The PMLE for common HR in matched-pair cohort studies can be expressed by a simple formula based on only two numbers: the number of pairs in which the exposed has a shorter observed time and experienced an event (*G*) and the number of pairs in which the unexposed has a shorter observed time and experienced an event (*H*). Such a simple form of HR estimators is unique to PMLE. Corresponding Poisson rate regression may be a stratified Poisson model with common HR, with the likelihood conditional on the total number of events in each stratum (0, 1, or 2), which reduces to binomial likelihood [34]. The CMLE for common HR is the solution of $\sum\nolimits_{k} {\frac{{Y_{k1} X_{k0} - {\text{HR}} \cdot Y_{k0} X_{k1} }}{{X_{k0} + {\text{HR}} \cdot X_{k1} }}} = 0$, which is dependent on observed time *X*. It is slightly different from the Mantel–Haenszel rate ratio estimator, $\frac{{\sum\nolimits_{k} {{{Y_{k1} X_{k0} } \mathord{\left/ {\vphantom {{Y_{k1} X_{k0} } {(X_{k0} + X_{k1} )}}} \right. \kern-0pt} {(X_{k0} + X_{k1} )}}} }}{{\sum\nolimits_{k} {{{Y_{k0} X_{k1} } \mathord{\left/ {\vphantom {{Y_{k0} X_{k1} } {(X_{k0} + X_{k1} )}}} \right. \kern-0pt} {(X_{k0} + X_{k1} )}}} }}$, which approximates the Poisson CMLE around HR = 1.

The current simple expression of stratified PMLE and its relationship with C_τ,pair_ is also unique to a binary exposure. We could not find any simple expression of estimators of multiple effect-parameters for more than 2 exposure levels, or even a single parameter in the stratified Cox model (i.e., linear effect on log-hazard) for continuous exposure, say, *V*. In the latter case, an adequate definition of matched-pair overall C-index may be C_τ,pair_ = Pr(*V*_*k*1_ > *V*_*k*2_| *T*_*k*1_ < *T*_*k*2_, *T*_*k*1_ < *τ*) (if log-HR *β* > 0). This may be estimated by redefining a “binary” exposure *E*, such that *E*_*k*1_ = *I*(*V*_*k*1_ > *V*_*k*2_) and *E*_*k*2_ = *I*(*V*_*k*1_ < *V*_*k*2_), and calculating *G*/(*G* + *H*) as if the dataset comes from a pair-matched cohort. However, the limiting value of this statistic is now dependent on the censoring distribution irrespective of the underlying model form. Instead, the stratified PMLE obtained by iterative maximization (e.g., by Newton–Raphson algorithm) of partial likelihood may be used to estimate overall-C; following the similar discussion of Gönen and Heller [35], the average of $\frac{{\exp (\hat{\beta } \cdot \left| {V_{k1} - V_{k2} } \right|)}}{{1 + \exp (\hat{\beta } \cdot \left| {V_{k1} - V_{k2} } \right|)}}$ across *n* matched pairs converges to Pr(*V*_*k*1_ > *V*_*k*2_| *T*_*k*1_ < *T*_*k*2_, *T*_*k*1_ < *τ*) if the assumed linear-effect Cox model is correct and if no tie-event occurs [36]. Although the simple expression of PMLE is not applicable to continuous/multiple-level exposures, the stratified PMLE is still relevant to interpretation of a matched-pair C-index for time-to-event outcomes.

It is well recognized that whenever matching variables in case–control studies are associated with either exposure or disease in an original cohort, unless exposure effect on disease is absent, they must be adjusted in analysis irrespective of whether they are confounders [1, 37]. Unlike case–control studies, ignoring matching in cohort studies generally produce valid point estimates when the matching ratio is constant across strata and no censoring occurs [32, 37]. This phenomenon is due to the design that completely balances the matched variables between exposed and unexposed groups. In the theory of causal diagrams, design unfaithfulness occurs, i.e., exposure and matching variable are independent in the matched subpopulation despite being connected in the causal diagram [37–39]. However, when additional confounders are adjusted in the analyses, such cancellation breaks down and ignoring matching variables results in biased estimates [32]. Moreover, as shown in our simulation, if the proportionality of hazards holds given matching variables and if censoring is present, the estimated HR under marginal models would be biased away from both conditional HR and an “average” of time-varying HR [19]. The bias depends on the censoring rate even if events are independently censored. Similar to this phenomenon, if the matching variable is a risk factor for outcome and competing risks or losses to follow-up are associated with both exposure and the matching variable, estimates not adjusting for the matching variables are no longer unbiased even in the null exposure effect [1, 37]. Our simulation also showed that censorship dependent on the exposure and the matched pair invalidates the marginal estimate and statistical test.

Matching is often conducted in analysis stage, especially with estimated propensity scores in order to reduce confounding, as in our real data example. Contrary to actually matched data, analytical subtleties in propensity-matching have been discussed in recent literature. First, whether propensity-matching should adjust for as sampling variation remains controversial [18, 40]. Second, conditional effect parameters are usually not targeted in propensity-matching because conditioning on propensity-matched pairs has little interpretability. Third, at the cost of balancing between exposure groups, propensity-matching discards some proportion of available data. If one is interested in the effect on the exposed (the marginal effect-measure if 1 unexposed is matched on 1 exposed), one can expect more efficient estimates are obtained by differential propensity-weighting for exposed and unexposed groups [41] than marginal modeling with robust variance estimator after propensity-matching. While weighting directly uses the estimated propensity scores from fitted models [42, 43], matching only uses the ranks of estimated propensity scores within an allowable caliper width. As ranks are less sensitive to misspecification of the model form, one may argue the propensity-matching analyses are more robust than estimates using propensity-weighting or outcome-regression, or both [44]. Detailed investigation of this bias–variance trade-off between propensity-matching and weighting for marginal estimates is interesting future work. From these viewpoints, however, a PML estimator of common HR may be of little use along with propensity-matching.

## Conclusion

Although common HR itself may have limited value in public health literature because of its noncollapsibility and built-in selection bias [19], the simple and intuitive representation of its estimator would be a useful summary of the exposure effect. The common HR estimator may be a good alternative to the marginal HR estimators if loss-to-follow-up is not negligible and/or depends on exposure and matching variables. Otherwise, survival time or risk comparisons should be used to overcome the problems with causal interpretation of HR [17, 19].

## Appendix 1: PMLE with tied-event pairs

Table 6 shows the additional types of pairs when tied events occur in the same pair. By convention, when censoring and event simultaneously occur in the data, that pair is treated as if censoring was measured after event: types 10 and 11 (Table 6) are treated as types 3 and 4 (Table 2), respectively. Then, we only have to consider how to treat tie-event (type 12) by each method: the exact, Breslow’s, or Efron’s methods are commonly used [11, 12].

**Table 6:** Pair types with tied data with at least one event

Type	Number of pairs	Observed data in the pair	Observed time	*Y*_*k*1_	*Y*_*k*0_
10	*n*_10_	Exposed gets event and unexposed censored simultaneously	*X*_*k*1_ = *X*_*k*0_	1	0
11	*n*_11_	Unexposed gets event and exposed censored simultaneously	*X*_*k*1_ = *X*_*k*0_	0	1
12	*n*_12_	Exposed and unexposed get events simultaneously	*X*_*k*1_ = *X*_*k*0_	1	1

If tie-events are treated by the exact method, the contribution of that type of pair is exp(*β*)/{1 + exp(*β*)} + 1/{1 + exp(*β*)} = 1, which means these pairs do not contribute the partial-likelihood. Thus, we only have to modify the matched-pair common HR estimator as *G*/*H* in which type 12-pairs are counted in neither *G* nor *H*: *G* = *n*_1_ + *n*_3_ + *n*_10_ and *H* = *n*_2_ + *n*_4_ + *n*_11_. On the contrary, if tie-events are treated by Breslow’s methods, the contribution of that pair type is exp(*β*)/{1 + exp(*β*)}^2^, or by Efron’s methods, 2 × exp(*β*)/{1 + exp(*β*)}^2^. This means that the partial likelihood counts type 12-pairs in both *G* and *H*: a modified matched-pair common HR estimator corresponding Breslow’s or Efron’s methods is *G*/*H* in which type 12-pairs are counted in both *G* and *H* (i.e., *G* = *n*_1_ + *n*_3_ + *n*_10_ + *n*_12_ and *H* = *n*_2_ + *n*_4_ + *n*_11_ + *n*_12_).

We provide in Table 7 a typical dataset (50 pairs) generated with true HR = 2 with independent censoring (rate = 1) in the main text. To view the impact of tied event data, we rounded the observed time to one decimal place. Among the comparable pairs, *n*_1_ + *n*_3_ + *n*_10_ = 18, *n*_2_ + *n*_4_ + *n*_11_ = 7 and *n*_12_ = 1 (pair 30). Using exact tie-breaking method, *G* = 18 and *H* = 7; PMLE of common HR is *G*/*H* = 2.57 with 95% confidence limits exp{log(2.57) ± 1.96 × √(1/18 + 1/7)} = 1.07 and 6.16. Using Breslow’s and Efron’s tie-breaking method, *G* = 19 and *H* = 8 and the estimate (95% confidence limits) is 2.38 (1.04, 5.43). These data are in perfect accordance with the results obtained by fitting a Cox model stratified on matched-pairs via the PHREG procedure with options “ties = exact” and “ties = Breslow” (default in current version of SAS; “ties = Efron” provide the same result), respectively.

**Table 7:** Simulated 50 pairs generated with true HR = 2 with independent censoring (rate = 1)

Pair (*k*)	Exposure (*e*)	Time (*X*_ke_)	Event (*Y*_*ke*_)	Pair (*k*)	Exposure (*e*)	Time (*X*_ke_)	Event (*Y*_*ke*_)
1	0	0.1	0	26	0	1	0
1	1	0.8	1	26	1	0	0
2	0	0.3	0	27	0	0	1
2	1	0.3	0	27	1	0.2	1
3	0	0.6	0	28	0	0.1	0
3	1	0.1	0	28	1	0.2	1
4	0	0.2	0	29	0	0.4	0
4	1	0.2	1	29	1	0.3	0
5	0	0.2	0	30	0	0	1
5	1	0.4	1	30	1	0	1
6	0	1.6	0	31	0	0.1	1
6	1	0.5	0	31	1	0.5	1
7	0	0.4	1	32	0	0.2	0
7	1	0.2	0	32	1	1.1	0
8	0	1.1	1	33	0	0.6	0
8	1	0.4	1	33	1	0.4	1
9	0	1.2	0	34	0	0.2	1
9	1	0.1	0	34	1	0.1	1
10	0	0.3	1	35	0	1	0
10	1	0.2	0	35	1	0.3	0
11	0	0.6	1	36	0	2.7	1
11	1	1.3	1	36	1	0	0
12	0	0.1	1	37	0	0	0
12	1	0	1	37	1	0.2	1
13	0	0.3	0	38	0	0	0
13	1	0.1	0	38	1	1.3	0
14	0	0.1	0	39	0	0.4	1
14	1	0	1	39	1	0.1	1
15	0	0	1	40	0	0.8	0
15	1	0.1	1	40	1	0	1
16	0	0.5	0	41	0	0	1
16	1	0.3	1	41	1	0.1	1
17	0	2.7	0	42	0	0.1	0
17	1	0.6	1	42	1	0.1	1
18	0	0.2	0	43	0	2.4	0
18	1	0.3	0	43	1	1.3	1
19	0	1.5	0	44	0	0.7	1
19	1	0.2	0	44	1	0.2	1
20	0	0.1	0	45	0	0.2	1
20	1	0	1	45	1	0.1	1
21	0	0.5	1	46	0	0.3	0
21	1	0.7	0	46	1	0.4	1
22	0	0.4	1	47	0	0.1	0
22	1	0.2	1	47	1	0.1	0
23	0	0	0	48	0	0.1	1
23	1	0.3	0	48	1	0.2	1
24	0	0.2	0	49	0	0.5	1
24	1	0.3	1	49	1	0	0
25	0	1.9	1	50	0	0.5	1
25	1	0.1	1	50	1	0	1

## Appendix 2: Equivalence between the limiting value of *G*/*H* and C_*τ*_ with censoring under stratified proportional hazards model

Following the main text, the C*τ* estimator *G*/(*G* + *H*) is expressed as

$$\frac{{\sum\nolimits_{k = 1}^{n} {Y_{k1} I(X_{k1} < X_{k0} )} }}{{\sum\nolimits_{k = 1}^{n} {Y_{k1} I(X_{k1} < X_{k0} ) + Y_{k0} I(X_{k1} > X_{k0} )} }},$$

where *I*(A) is the indicator function (1 if A is true, 0 otherwise). Because *Y*_*k*e_ = *I*{*T*_*k*1_ < min(*U*_*ke*_, *τ*)}, it is rewritten as

$$\begin{aligned} \frac{{\sum\nolimits_{k = 1}^{n} {I\{ T_{k1} < \hbox{min} (U_{k1} ,T_{k0} ,U_{k0} ,\tau )\} } }}{{\sum\nolimits_{k = 1}^{n} {I\{ T_{k1} < \hbox{min} (U_{k1} ,T_{k0} ,U_{k0} ,\tau )\} + I\{ T_{k0} < \hbox{min} (U_{k0} ,T_{k1} ,U_{k1} ,\tau )\} } }} \hfill \\ = \frac{{\sum\nolimits_{k = 1}^{n} {\sum\nolimits_{{(e_{1} ,e_{2} ) = (0,1),(1,0)}} {I\{ T_{{ke_{1} }} < \hbox{min} (U_{{ke_{1} }} ,T_{{ke_{2} }} ,U_{{ke_{2} }} ,\tau )\} I(e_{1} = 1,\quad e_{2} = 0{\text{ in pair }}k)} } }}{{\sum\nolimits_{k = 1}^{n} {\sum\nolimits_{{(e_{1} ,e_{2} ) = (0,1),(1,0)}} {I\{ T_{{ke_{1} }} < \hbox{min} (U_{{ke_{1} }} ,T_{{ke_{2} }} ,U_{{ke_{2} }} ,\tau )\} } } }} \hfill \\ \end{aligned}$$

Note that as the pair-number *n* increases, the denominator and the numerator divided by *n* ^2^ converges to $\Pr \{ T_{{ke_{1} }} < T_{{ke_{2} }} ,T_{{ke_{1} }} < \hbox{min} (U_{{ke_{1} }} ,U_{{ke_{2} }} ,\tau )\}$ and $\Pr \{ T_{{ke_{1} }} < T_{{ke_{2} }} ,T_{{ke_{1} }} < \hbox{min} (U_{{ke_{1} }} ,U_{{ke_{2} }} ,\tau ),e_{1} = 1,\quad e_{2} = 0\}$, respectively [15]. Therefore, with drop-out before the end of follow-up *τ*, *G*/(*G* + *H*) converges to conditional probability (3),$\Pr \{ e_{1} = 1,e_{2} = 0{\text{ in pair }}k\left| {T_{{ke_{1} }} < T_{{ke_{2} }} ,T_{{ke_{1} }} < \hbox{min} (U_{{ke_{1} }} ,U_{{ke_{2} }} ),T_{{ke_{1} }} < \tau } \right.\}$.The limiting value of *G*/(*G* + *H*) is

$$\begin{array}{*{20}l} {\Pr \{ T_{k1} < T_{k0} |T_{k1} < \hbox{min} (U_{k0} ,U_{k1} ,\tau ){\text{ or}} T_{k0} < \hbox{min} (U_{k0} ,U_{k1} ,\tau )\} } \hfill \\ { = [\Pr \{ T_{k1} < T_{k0} ,T_{k1} < \hbox{min} (U_{k0} ,U_{k1} ,\tau )\} + \Pr \{ T_{k1} < T_{k0} ,T_{k0} < \hbox{min} (U_{k0} ,U_{k1} ,\tau )\} } \hfill \\ \begin{aligned} {-}\Pr \{ T_{k1} < T_{k0} ,T_{k1} < \hbox{min} (U_{k0} ,U_{k1} ,\tau ),T_{k0} < \hbox{min} (U_{k0} ,U_{k1} ,\tau )\} ]/\Pr \{ T_{k1} < \hbox{min} (U_{k0} ,U_{k1} ,\tau ){\text{ or}} T_{k0} < \hbox{min} (U_{k0} ,U_{k1} ,\tau )\} \hfill \\ = \Pr \{ T_{k1} < T_{k0} ,T_{k1} < \hbox{min} (U_{k0} ,U_{k1} ,\tau )\} /\Pr \{ T_{k1} < \hbox{min} (U_{k0} ,U_{k1} ,\tau ){\text{ or}}\,T_{k0} < \hbox{min} (U_{k0} ,U_{k1} ,\tau )\} . \hfill \\ \end{aligned} \hfill \\ \end{array}$$

If *U*_*k*0_ and *U*_*k*1_ are independent of each other and are independent of both *T*_*k*1_ and *T*_*k*0_ within all matched-pair *k*, Pr{*T*_*k*1_ < *T*_*k*0_, *T*_*k*1_ < min(*U*_*k*0_, *U*_*k*1_, *τ*)} is

$$\Pr (T_{k1} < T_{k0} ,T_{k1} < U_{k0} ,T_{k1} < U_{k1} ,T_{k1} < \tau ) = \int_{0}^{\tau } {f_{k1} (t)S_{k0} (t)S_{{U_{k1} }} (t)S_{{U_{k0} }} (t)dt} ,$$

where *S*_*Uke*_(*t*) is a survival function of *U*_*ke*_. Similarly, the limiting value of *H*/(*G* + *H*) is Pr{*T*_*k*1_ > *T*_*k*0_ | *T*_*k*1_ < min(*U*_*k*0_, *U*_*k*1_, *τ*) or *T*_*k*0_ < min(*U*_*k*0_, *U*_*k*1_, *τ*)} = Pr{*T*_*k*1_ > *T*_*k*0_, *T*_*k*0_ < min(*U*_*k*0_, *U*_*k*1_, *τ*)}/Pr{*T*_*k*1_ < min(*U*_*k*0_, *U*_*k*1_, *τ*) or *T*_*k*0_ < min(*U*_*k*0_, *U*_*k*1_, *τ*)} in which the numerator is $\int_{0}^{\tau } {f_{k0} (t)S_{k1} (t)S_{{U_{k1} }} (t)S_{{U_{k0} }} (t)dt}$. Suppose stratified proportional hazards assumption (1) holds. The limit of ratio of *G*/(*G* + *H*) and *H*/(*G* + *H*), or *G*/*H*, is then

$$\frac{{\int_{0}^{\tau } {f_{k1} (t)S_{k0} (t)S_{{U_{k1} }} (t)S_{{U_{k0} }} (t)dt} }}{{\int_{0}^{\tau } {f_{k0} (t)S_{k1} (t)S_{{U_{k1} }} (t)S_{{U_{k0} }} (t)dt} }} = \frac{{\int_{0}^{\tau } {\lambda_{k0} (t){\text{e}}^{\beta } S_{k1} (t)S_{k0} (t)S_{{U_{k1} }} (t)S_{{U_{k0} }} (t)dt} }}{{\int_{0}^{\tau } {\lambda_{k0} (t)S_{k0} (t)S_{k1} (t)S_{{U_{k1} }} (t)S_{{U_{k0} }} (t)dt} }} = {\text{e}}^{\beta } ,$$

which is equal to C_*τ*,pair_/(1 − C_*τ*,pair_) by following the argument in the main text.

## Additional file

**Additional file 1.** Additional simulation results and SAS codes for simulation and for matching women based on estimated propensity scores in the Rotterdam tumor database.

## Abbreviations

CMLE: Conditional maximum likelihood estimator
HR: Hazard ratio
OR: Odds ratio
PMLE: Partial maximum likelihood estimator
