# Supplementary material for: On hazard ratio estimators by proportional hazards models in matched-pair cohort studies
Source: Emerg Themes Epidemiol. 2017 Jun 5;14:6. doi: 10.1186/s12982-017-0060-8 (PMC5460539; doi:10.1186/s12982-017-0060-8)
Supplement: Supplementary file 1 — Additional file 1. Additional simulation results and SAS codes for simulation and for matching women based on estimated propensity scores in the Rotterdam tumor database. [file 12982_2017_60_MOESM1_ESM.docx]

**Additional file to “On hazard ratio estimators in proportional hazards models in matched-pair cohort studies”**

Tomohiro Shinozaki, Mohammad Ali Mansournia, and Yutaka Matsuyama

(Date: Feb 13, 2017)

1. **SAS program for simulation data presented in the main text (exponential model)**

/* Data are generated in SAS version 9.4 */

/* 1. For independent censoring scenarios */

**%macro** sim(nsamp=**2000**, npair=, beta=, censrate=);

data sim;

call streaminit(**20140723**);

do samp = **1** to &nsamp;

do i = **1** to &npair;

alpha = rand("Normal");

do e = **0**, **1**;

u = rand("Uniform");

err = log(-log(**1** - u));

t = exp(alpha - e*&beta + err);

u2 = rand("Uniform");

err2 = log(-log(**1** - u2));

c = exp(-log(&censrate) + err2);

/* NOTE: Equivalent to: c = rand("exponential")/&censrate */

tobs = min(t, c);

d = (tobs = t);

output;

end;

end;

end;

run;

**%mend**;

/* ----------------------------------------------------------------

All combinations were examined in the simulation studies.

npair: Number of pairs in one dataset. (npair = 50 or 250)

beta: True conditional log hazard ratio. (beta = log(2), log(1), or log(0.5))

censrate: Rate parameter in exponential distribution for independent censoring. (censrate = 1, 2, or 4)

---------------------------------------------------------------- */

/* 2. For dependent but conditionally independent censoring scenarios */

**%macro** sim2(nsamp=**2000**, npair=, beta=, censrateratio=);

data sim;

call streaminit(**20140723**);

do samp = **1** to &nsamp;

do i = **1** to &npair;

alpha = rand("Normal");

do e = **0**, **1**;

u = rand("Uniform");

err = log(-log(**1** - u));

t = exp(alpha - e*&beta + err);

u2 = rand("Uniform");

err2 = log(-log(**1** - u2));

c = exp(alpha - e*&censrateratio + err2);

tobs = min(t, c);

d = (tobs = t);

output;

end;

end;

end;

run;

**%mend**;

/* ----------------------------------------------------------------

All combinations were examined in the simulation studies.

npair: Number of pairs in one dataset. (npair = 50 or 250)

beta: True conditional log hazard ratio. (beta = log(2), log(1), or log(0.5))

censrateratio: Log rate ratio for censoring by exposure. (censrateratio = log(0.25), log(1), or log(4))

---------------------------------------------------------------- */

/* 3. Define the marginal parameters */

**%macro** mHR(npair=**5000000**, beta=);

%global m_beta;

data m;

call streaminit(**20150223**);

do i = **1** to &npair;

alpha = rand("Normal");

do e = **0**, **1**;

u = rand("Uniform");

err = log(-log(**1** - u));

t = exp(alpha - e*&beta + err);

d = **1**;

output;

end;

end;

run;

ods listing close;

ods output ParameterEstimates = m_param;

proc phreg data=m;

model t*d(**0**) = e;

run;

ods listing;

data m_param;

set m_param;

call symput("m_beta", Estimate);

run;

**%mend**;

/* ----------------------------------------------------------------

“True” marginal hazard ratio is stored in a macro variable &m_beta.

---------------------------------------------------------------- */

1. **Simulation results for *n* = 50**

Supplementary Table 1. Simulated estimates for different Cox models with independent censoring, varying censoring rate (2000 repetitions, *n* = 50).

|  | Censoring rate | MCSE | MESE | Log conditional-HR | | |  | Log marginal-HR | | |
| --- | --- | --- | --- | --- | --- | --- | --- | --- | --- | --- |
| Method |  |  |  | Bias | 95%CP (%) | RMSE |  | Bias | 95%CP (%) | RMSE |
| Log conditional-HR = log(2) = 0.693; Log marginal-HR = 0.437 | | | | | | | | | | |
| Frailty Cox model | 1 | 0.30 | 0.30 | -0.01 | 95.61 | 0.30 |  | 0.25 | 88.36 | 0.39 |
| Stratified Cox model |  | 0.40 | 0.40 | 0.02 | 97.10 | 0.40 |  | 0.28 | 92.95 | 0.48 |
| Unstratified Cox model without sandwich variance |  | 0.23 | 0.27 | -0.18 | 93.50 | 0.29 |  | 0.08 | 97.65 | 0.24 |
| Unstratified Cox model with sandwich variance |  | 0.23 | 0.23 | -0.18 | 86.55 | 0.29 |  | 0.08 | 94.05 | 0.24 |
|  |  |  |  |  |  |  |  |  |  |  |
| Frailty Cox model | 2 | 0.33 | 0.34 | -0.01 | 96.09 | 0.33 |  | 0.24 | 90.85 | 0.41 |
| Stratified Cox model |  | 0.47 | 0.47 | 0.04 | 97.40 | 0.47 |  | 0.29 | 94.45 | 0.55 |
| Unstratified Cox model without sandwich variance |  | 0.27 | 0.32 | -0.15 | 95.85 | 0.31 |  | 0.11 | 96.95 | 0.29 |
| Unstratified Cox model with sandwich variance |  | 0.27 | 0.27 | -0.15 | 90.35 | 0.31 |  | 0.11 | 93.95 | 0.29 |
|  |  |  |  |  |  |  |  |  |  |  |
| Frailty Cox model | 4 | 0.40 | 0.41 | -0.01 | 96.46 | 0.40 |  | 0.24 | 94.05 | 0.47 |
| Stratified Cox model |  | 0.58 | 0.58 | 0.05 | 97.44 | 0.58 |  | 0.30 | 96.74 | 0.65 |
| Unstratified Cox model without sandwich variance |  | 0.34 | 0.39 | -0.12 | 96.95 | 0.36 |  | 0.14 | 97.55 | 0.37 |
| Unstratified Cox model with sandwich variance |  | 0.34 | 0.34 | -0.12 | 93.25 | 0.36 |  | 0.14 | 94.65 | 0.37 |
|  |  |  |  |  |  |  |  |  |  |  |
| Log conditional-HR = log(1) = 0; Log marginal-HR = 0 | | | | | | | | | | |
| Frailty Cox model | 1 | 0.30 | 0.31 | 0.00 | 96.56 | 0.30 |  | 0.00 | 96.56 | 0.30 |
| Stratified Cox model |  | 0.39 | 0.41 | 0.01 | 96.30 | 0.39 |  | 0.01 | 96.30 | 0.39 |
| Unstratified Cox model without sandwich variance |  | 0.24 | 0.29 | 0.00 | 98.65 | 0.24 |  | 0.00 | 98.65 | 0.24 |
| Unstratified Cox model with sandwich variance |  | 0.24 | 0.24 | 0.00 | 95.00 | 0.24 |  | 0.00 | 95.00 | 0.24 |
|  |  |  |  |  |  |  |  |  |  |  |
| Frailty Cox model | 2 | 0.34 | 0.37 | 0.01 | 96.86 | 0.34 |  | 0.01 | 96.86 | 0.34 |
| Stratified Cox model |  | 0.48 | 0.49 | 0.02 | 97.20 | 0.48 |  | 0.02 | 97.20 | 0.48 |
| Unstratified Cox model without sandwich variance |  | 0.29 | 0.34 | 0.01 | 98.50 | 0.29 |  | 0.01 | 98.50 | 0.29 |
| Unstratified Cox model with sandwich variance |  | 0.29 | 0.30 | 0.01 | 95.45 | 0.29 |  | 0.01 | 95.45 | 0.29 |
|  |  |  |  |  |  |  |  |  |  |  |
| Frailty Cox model | 4 | 0.43 | 0.45 | 0.00 | 97.20 | 0.43 |  | 0.00 | 97.20 | 0.43 |
| Stratified Cox model |  | 0.62 | 0.62 | 0.01 | 97.44 | 0.62 |  | 0.01 | 97.44 | 0.62 |
| Unstratified Cox model without sandwich variance |  | 0.38 | 0.43 | 0.01 | 98.30 | 0.38 |  | 0.01 | 98.30 | 0.38 |
| Unstratified Cox model with sandwich variance |  | 0.38 | 0.38 | 0.01 | 96.60 | 0.38 |  | 0.01 | 96.60 | 0.38 |
|  |  |  |  |  |  |  |  |  |  |  |
| Log conditional-HR = log(0.5) = –0.693; Log marginal-HR = –0.438 | | | | | | | | | | |
| Frailty Cox model | 1 | 0.33 | 0.34 | 0.02 | 95.88 | 0.33 |  | -0.23 | 92.38 | 0.40 |
| Stratified Cox model |  | 0.47 | 0.47 | -0.02 | 97.05 | 0.47 |  | -0.28 | 95.10 | 0.54 |
| Unstratified Cox model without sandwich variance |  | 0.27 | 0.32 | 0.16 | 94.85 | 0.31 |  | -0.09 | 97.60 | 0.28 |
| Unstratified Cox model with sandwich variance |  | 0.27 | 0.27 | 0.16 | 90.15 | 0.31 |  | -0.09 | 94.90 | 0.28 |
|  |  |  |  |  |  |  |  |  |  |  |
| Frailty Cox model | 2 | 0.39 | 0.41 | 0.03 | 96.63 | 0.39 |  | -0.23 | 94.12 | 0.45 |
| Stratified Cox model |  | 0.58 | 0.58 | -0.04 | 97.65 | 0.58 |  | -0.30 | 97.10 | 0.65 |
| Unstratified Cox model without sandwich variance |  | 0.34 | 0.39 | 0.13 | 96.95 | 0.36 |  | -0.13 | 97.55 | 0.36 |
| Unstratified Cox model with sandwich variance |  | 0.34 | 0.34 | 0.13 | 93.05 | 0.36 |  | -0.13 | 95.00 | 0.36 |
|  |  |  |  |  |  |  |  |  |  |  |
| Frailty Cox model | 4 | 0.50 | 0.52 | 0.02 | 96.88 | 0.50 |  | -0.24 | 95.70 | 0.56 |
| Stratified Cox model |  | 0.71 | 0.74 | -0.02 | 97.48 | 0.71 |  | -0.28 | 98.82 | 0.76 |
| Unstratified Cox model without sandwich variance |  | 0.61 | 2.37 | 0.07 | 97.35 | 0.61 |  | -0.18 | 98.15 | 0.63 |
| Unstratified Cox model with sandwich variance |  | 0.61 | 0.45 | 0.07 | 94.55 | 0.61 |  | -0.18 | 95.95 | 0.63 |

MCSE, empirical (Monte Carlo) standard error; MESE, mean estimated standard error; 95%CP, coverage proportion of 95% confidence interval; RMSE, root mean square error.

Supplementary Table 2. Simulated estimates for different Cox models with conditionally independent censoring given matched-pair and exposure, varying censoring rate ratio by exposure (2,000 Repetitions, *n* = 50).

|  | Censoring rate ratio by exposure | MCSE | MESE | Log conditional-HR | | |  | Log marginal-HR | | |
| --- | --- | --- | --- | --- | --- | --- | --- | --- | --- | --- |
| Method |  |  |  | Bias | 95%CP (%) | RMSE |  | Bias | 95%CP (%) | RMSE |
| Log conditional-HR = log(2) = 0.693; Log marginal-HR = 0.437 | | | | | | | | | | |
| Frailty Cox model | 0.25 | 0.28 | 0.28 | 0.10 | 94.40 | 0.29 |  | 0.36 | 77.12 | 0.45 |
| Stratified Cox model |  | 0.36 | 0.37 | 0.02 | 96.30 | 0.36 |  | 0.27 | 92.00 | 0.45 |
| Unstratified Cox model without sandwich variance |  | 0.21 | 0.26 | -0.03 | 98.10 | 0.21 |  | 0.22 | 91.70 | 0.31 |
| Unstratified Cox model with sandwich variance |  | 0.21 | 0.22 | -0.03 | 94.75 | 0.21 |  | 0.22 | 83.85 | 0.31 |
|  |  |  |  |  |  |  |  |  |  |  |
| Frailty Cox model | 1 | 0.29 | 0.29 | 0.00 | 95.72 | 0.29 |  | 0.26 | 87.25 | 0.39 |
| Stratified Cox model |  | 0.40 | 0.40 | 0.02 | 95.95 | 0.40 |  | 0.28 | 93.00 | 0.49 |
| Unstratified Cox model without sandwich variance |  | 0.23 | 0.27 | -0.13 | 94.90 | 0.27 |  | 0.12 | 96.85 | 0.26 |
| Unstratified Cox model with sandwich variance |  | 0.23 | 0.23 | -0.13 | 90.30 | 0.27 |  | 0.12 | 92.90 | 0.26 |
|  |  |  |  |  |  |  |  |  |  |  |
| Frailty Cox model | 4 | 0.36 | 0.36 | -0.24 | 90.73 | 0.43 |  | 0.01 | 96.31 | 0.36 |
| Stratified Cox model |  | 0.51 | 0.52 | 0.04 | 97.85 | 0.51 |  | 0.30 | 96.39 | 0.59 |
| Unstratified Cox model without sandwich variance |  | 0.30 | 0.34 | -0.38 | 83.65 | 0.48 |  | -0.12 | 96.50 | 0.33 |
| Unstratified Cox model with sandwich variance |  | 0.30 | 0.30 | -0.38 | 76.45 | 0.48 |  | -0.12 | 94.05 | 0.33 |
|  |  |  |  |  |  |  |  |  |  |  |
| Log conditional-HR = log(1) = 0; Log marginal-HR = 0 | | | | | | | | | | |
| Frailty Cox model | 0.25 | 0.27 | 0.28 | 0.15 | 92.95 | 0.31 |  | 0.15 | 92.95 | 0.31 |
| Stratified Cox model |  | 0.35 | 0.37 | 0.01 | 97.20 | 0.35 |  | 0.01 | 97.20 | 0.35 |
| Unstratified Cox model without sandwich variance |  | 0.22 | 0.26 | 0.17 | 94.75 | 0.28 |  | 0.17 | 94.75 | 0.28 |
| Unstratified Cox model with sandwich variance |  | 0.22 | 0.22 | 0.17 | 89.15 | 0.28 |  | 0.17 | 89.15 | 0.28 |
|  |  |  |  |  |  |  |  |  |  |  |
| Frailty Cox model | 1 | 0.29 | 0.31 | 0.00 | 96.34 | 0.29 |  | 0.00 | 96.34 | 0.29 |
| Stratified Cox model |  | 0.39 | 0.41 | 0.01 | 97.15 | 0.39 |  | 0.01 | 97.15 | 0.39 |
| Unstratified Cox model without sandwich variance |  | 0.25 | 0.29 | 0.00 | 98.35 | 0.25 |  | 0.00 | 98.35 | 0.25 |
| Unstratified Cox model with sandwich variance |  | 0.25 | 0.25 | 0.00 | 95.60 | 0.25 |  | 0.00 | 95.60 | 0.25 |
|  |  |  |  |  |  |  |  |  |  |  |
| Frailty Cox model | 4 | 0.41 | 0.41 | -0.27 | 93.15 | 0.49 |  | -0.27 | 93.15 | 0.49 |
| Stratified Cox model |  | 0.55 | 0.56 | 0.02 | 97.70 | 0.55 |  | 0.02 | 97.70 | 0.55 |
| Unstratified Cox model without sandwich variance |  | 0.37 | 0.40 | -0.32 | 92.75 | 0.50 |  | -0.32 | 92.75 | 0.50 |
| Unstratified Cox model with sandwich variance |  | 0.37 | 0.37 | -0.32 | 89.05 | 0.50 |  | -0.32 | 89.05 | 0.50 |
|  |  |  |  |  |  |  |  |  |  |  |
| Log conditional-HR = log(0.5) = –0.693; Log marginal-HR = –0.438 | | | | | | | | | | |
| Frailty Cox model | 0.25 | 0.30 | 0.30 | 0.18 | 90.49 | 0.35 |  | -0.07 | 95.27 | 0.31 |
| Stratified Cox model |  | 0.41 | 0.42 | -0.02 | 96.80 | 0.41 |  | -0.28 | 94.05 | 0.49 |
| Unstratified Cox model without sandwich variance |  | 0.24 | 0.28 | 0.34 | 80.45 | 0.42 |  | 0.09 | 97.15 | 0.25 |
| Unstratified Cox model with sandwich variance |  | 0.24 | 0.24 | 0.34 | 70.00 | 0.42 |  | 0.09 | 93.80 | 0.25 |
|  |  |  |  |  |  |  |  |  |  |  |
| Frailty Cox model | 1 | 0.33 | 0.35 | 0.00 | 96.60 | 0.33 |  | -0.25 | 91.88 | 0.42 |
| Stratified Cox model |  | 0.47 | 0.48 | -0.03 | 97.10 | 0.47 |  | -0.29 | 95.40 | 0.55 |
| Unstratified Cox model without sandwich variance |  | 0.29 | 0.33 | 0.10 | 97.10 | 0.30 |  | -0.16 | 96.50 | 0.33 |
| Unstratified Cox model with sandwich variance |  | 0.29 | 0.29 | 0.10 | 94.25 | 0.30 |  | -0.16 | 93.85 | 0.33 |
|  |  |  |  |  |  |  |  |  |  |  |
| Frailty Cox model | 4 | 0.53 | 0.52 | -0.31 | 96.02 | 0.61 |  | -0.56 | 88.07 | 0.77 |
| Stratified Cox model |  | 0.66 | 0.68 | -0.04 | 98.27 | 0.67 |  | -0.30 | 98.32 | 0.73 |
| Unstratified Cox model without sandwich variance |  | 0.80 | 2.84 | -0.35 | 97.50 | 0.87 |  | -0.60 | 88.80 | 1.00 |
| Unstratified Cox model with sandwich variance |  | 0.80 | 0.48 | -0.35 | 96.00 | 0.87 |  | -0.60 | 85.40 | 1.00 |

MCSE, empirical (Monte Carlo) standard error; MESE, mean estimated standard error; 95%CP, coverage proportion of 95% confidence interval; RMSE, root mean square error.

1. **Additional simulation studies under non-constant baseline hazards (Weibull model)**

Instead of using the time-constant hazard *λ*_0_ (= 1) in the main text, Weibull time-to-event variables were used to emulate increasing or decreasing baseline hazards. The Baseline Weibull distribution with the shape parameter *k* and the scale parameter *σ* has the hazard function *λ*_0_(*t*) = *k*⋅*t^k^*^–1^(1/*σ*)*^k^*. From the proportional hazards model *λ_ke_*(*t*) = *λ*_0_(*t*)exp(*γ_k_*)exp(*β*⋅*e*), the hazard function for a member *e* in the pair *k* is expressed as *λ_ke_*(*t*) = *k*⋅*t^k^*^–1^[*σ*⋅exp{– (*γ_k_ + β*⋅*e*)/*k*}]^–^*^k^*. Hence, we generated random time-to-event data for member *e* in the pair *k* from Weibull distribution with the shape parameter *k* and the scale parameter *σ*⋅exp{– (*γ_k_ + β*⋅*e*)/*k*} (instead of the baseline scale parameter *σ*). In the series of simulations, *σ* was set at 1, and the two additional scenarios considered were: 1) all pairs have the same shape parameter *k*, namely, *k* = 2 (increasing hazard against follow-up time) or 0.5 (decreasing hazard); and 2) each pair has a distinct shape parameter *k*, the square of which was generated by standard log-normal distribution. All other parameters, as well as censoring distributions, were varied according to the main text (except for the number of pairs; only *n* = 250 was assessed).

- 1. **SAS program for generating data**

/* Additional Scenario 1: increasing or decreasing baseline hazard */

data sim;

call streaminit(**20170209**);

do samp = **1** to &nsamp;

do i = **1** to &npair;

alpha = rand("Normal");

do e = **0**, **1**;

t = rand("Weibull", &shape, exp(-(e*&beta + alpha)/&shape) );

c = rand("Weibull", **1**, **1**/&censrate);

/* NOTE: This is for independent censoring situations. To generate exposure-dependent censoring, replace the rand() generator by

c = rand("Weibull", 1, exp(-(e*&censrateratio + alpha)) )*/

tobs = min(t, c);

d = (tobs = t);

output;

end;

end;

end;

run;

/* Additional Scenario 2: varying Weibull shape parameter across pairs */

data sim;

call streaminit(**20170209**);

do samp = **1** to &nsamp;

do i = **1** to &npair;

alpha = rand("Normal");

shape = sqrt(exp(rand("Normal")));

do e = **0**, **1**;

t = rand("Weibull", shape, exp(-(alpha + e*&beta)/ shape) );

c = rand("Weibull", **1**, **1**/&censrate);

/* NOTE: This is for independent censoring situations. To generate exposure-dependent censoring, replace the rand() generator by

c = rand("Weibull", 1, exp(-(e*&censrateratio + alpha)) )*/

tobs = min(t, c);

d = (tobs = t);

output;

end;

end;

end;

run;

- 1. **Additional simulation results**

Supplementary Table 3. Simulation results from Additional Scenario 1-1: increasing baseline hazard with independent censoring (2000 repetitions, *n* = 250).

|  | Censoring rate | MCSE | MESE | Log conditional-HR | | |  | Log marginal-HR | | |
| --- | --- | --- | --- | --- | --- | --- | --- | --- | --- | --- |
| Method |  |  |  | Bias | 95%CP (%) | RMSE |  | Bias | 95%CP (%) | RMSE |
| Log conditional-HR = log(2) = 0.693; Log marginal-HR = 0.438* | | | | | | | | | | |
| Frailty Cox model | 1 | 0.15 | 0.15 | -0.03 | 93.89 | 0.15 |  | 0.23 | 67.38 | 0.27 |
| Stratified Cox model |  | 0.21 | 0.21 | 0.00 | 95.65 | 0.21 |  | 0.26 | 78.30 | 0.33 |
| Unstratified Cox model without sandwich variance |  | 0.11 | 0.13 | -0.20 | 67.10 | 0.23 |  | 0.05 | 95.95 | 0.13 |
| Unstratified Cox model with sandwich variance |  | 0.11 | 0.11 | -0.20 | 55.65 | 0.23 |  | 0.05 | 92.15 | 0.13 |
|  |  |  |  |  |  |  |  |  |  |  |
| Frailty Cox model | 2 | 0.19 | 0.19 | -0.04 | 94.57 | 0.19 |  | 0.21 | 79.60 | 0.28 |
| Stratified Cox model |  | 0.30 | 0.29 | 0.01 | 94.25 | 0.30 |  | 0.26 | 86.85 | 0.40 |
| Unstratified Cox model without sandwich variance |  | 0.15 | 0.17 | -0.16 | 85.05 | 0.22 |  | 0.09 | 94.10 | 0.18 |
| Unstratified Cox model with sandwich variance |  | 0.15 | 0.15 | -0.16 | 79.80 | 0.22 |  | 0.09 | 90.65 | 0.18 |
|  |  |  |  |  |  |  |  |  |  |  |
| Frailty Cox model | 4 | 0.26 | 0.26 | -0.05 | 95.35 | 0.26 |  | 0.21 | 89.01 | 0.33 |
| Stratified Cox model |  | 0.48 | 0.46 | 0.02 | 96.00 | 0.48 |  | 0.28 | 93.80 | 0.55 |
| Unstratified Cox model without sandwich variance |  | 0.24 | 0.24 | -0.12 | 93.25 | 0.26 |  | 0.13 | 93.45 | 0.27 |
| Unstratified Cox model with sandwich variance |  | 0.24 | 0.23 | -0.12 | 91.15 | 0.26 |  | 0.13 | 91.40 | 0.27 |
|  |  |  |  |  |  |  |  |  |  |  |
| Log conditional-HR = log(1) = 0; Log marginal-HR = 0* | | | | | | | | | | |
| Frailty Cox model | 1 | 0.15 | 0.15 | 0.00 | 95.04 | 0.15 |  | 0.00 | 95.04 | 0.15 |
| Stratified Cox model |  | 0.22 | 0.21 | 0.00 | 95.05 | 0.22 |  | 0.00 | 95.05 | 0.22 |
| Unstratified Cox model without sandwich variance |  | 0.12 | 0.13 | 0.00 | 97.50 | 0.12 |  | 0.00 | 97.50 | 0.12 |
| Unstratified Cox model with sandwich variance |  | 0.12 | 0.12 | 0.00 | 95.30 | 0.12 |  | 0.00 | 95.30 | 0.12 |
|  |  |  |  |  |  |  |  |  |  |  |
| Frailty Cox model | 2 | 0.19 | 0.20 | 0.00 | 96.27 | 0.19 |  | 0.00 | 96.27 | 0.19 |
| Stratified Cox model |  | 0.31 | 0.31 | 0.00 | 95.00 | 0.31 |  | 0.00 | 95.00 | 0.31 |
| Unstratified Cox model without sandwich variance |  | 0.16 | 0.18 | 0.00 | 96.90 | 0.16 |  | 0.00 | 96.90 | 0.16 |
| Unstratified Cox model with sandwich variance |  | 0.16 | 0.16 | 0.00 | 94.95 | 0.16 |  | 0.00 | 94.95 | 0.16 |
|  |  |  |  |  |  |  |  |  |  |  |
| Frailty Cox model | 4 | 0.29 | 0.29 | -0.01 | 95.30 | 0.29 |  | -0.01 | 95.30 | 0.29 |
| Stratified Cox model |  | 0.52 | 0.51 | -0.01 | 96.55 | 0.52 |  | -0.01 | 96.55 | 0.52 |
| Unstratified Cox model without sandwich variance |  | 0.26 | 0.27 | -0.01 | 96.05 | 0.26 |  | -0.01 | 96.05 | 0.26 |
| Unstratified Cox model with sandwich variance |  | 0.26 | 0.26 | -0.01 | 94.50 | 0.26 |  | -0.01 | 94.50 | 0.26 |
|  |  |  |  |  |  |  |  |  |  |  |
| Log conditional-HR = log(0.5) = –0.693; Log marginal-HR = –0.438* | | | | | | | | | | |
| Frailty Cox model | 1 | 0.16 | 0.16 | 0.04 | 94.93 | 0.17 |  | -0.22 | 74.26 | 0.27 |
| Stratified Cox model |  | 0.25 | 0.24 | 0.00 | 94.50 | 0.25 |  | -0.26 | 82.85 | 0.36 |
| Unstratified Cox model without sandwich variance |  | 0.13 | 0.14 | 0.18 | 76.00 | 0.23 |  | -0.07 | 94.80 | 0.15 |
| Unstratified Cox model with sandwich variance |  | 0.13 | 0.13 | 0.18 | 67.65 | 0.23 |  | -0.07 | 91.95 | 0.15 |
|  |  |  |  |  |  |  |  |  |  |  |
| Frailty Cox model | 2 | 0.21 | 0.22 | 0.04 | 95.10 | 0.22 |  | -0.22 | 84.80 | 0.30 |
| Stratified Cox model |  | 0.36 | 0.36 | -0.02 | 96.00 | 0.36 |  | -0.28 | 90.05 | 0.45 |
| Unstratified Cox model without sandwich variance |  | 0.19 | 0.20 | 0.14 | 90.10 | 0.23 |  | -0.12 | 93.50 | 0.22 |
| Unstratified Cox model with sandwich variance |  | 0.19 | 0.18 | 0.14 | 87.70 | 0.23 |  | -0.12 | 90.90 | 0.22 |
|  |  |  |  |  |  |  |  |  |  |  |
| Frailty Cox model | 4 | 0.33 | 0.33 | 0.02 | 95.22 | 0.33 |  | -0.23 | 91.25 | 0.40 |
| Stratified Cox model |  | 0.62 | 0.61 | -0.05 | 97.12 | 0.62 |  | -0.30 | 97.28 | 0.69 |
| Unstratified Cox model without sandwich variance |  | 0.31 | 0.31 | 0.08 | 95.05 | 0.32 |  | -0.18 | 93.55 | 0.35 |
| Unstratified Cox model with sandwich variance |  | 0.31 | 0.30 | 0.08 | 93.95 | 0.32 |  | -0.18 | 92.70 | 0.35 |

HR, hazard ratio; MCSE, empirical (Monte Carlo) standard error; MESE, mean estimated standard error; 95%CP, coverage proportion of 95% confidence interval; RMSE, root mean square error.

*Marginal hazard ratios were calculated under a large (*n* = 5 000 000 pairs) sample in each additional scenario.

Supplementary Table 4. Simulation results from Additional Scenario 1-2: with decreasing baseline hazard with independent censoring (2000 repetitions, *n* = 250).

|  | Censoring rate | MCSE | MESE | Log conditional-HR | | |  | Log marginal-HR | | |
| --- | --- | --- | --- | --- | --- | --- | --- | --- | --- | --- |
| Method |  |  |  | Bias | 95%CP (%) | RMSE |  | Bias | 95%CP (%) | RMSE |
| Log conditional-HR = log(2) = 0.693; Log marginal-HR = 0.438* | | | | | | | | | | |
| Frailty Cox model | 1 | 0.12 | 0.12 | -0.03 | 94.10 | 0.13 |  | 0.23 | 54.60 | 0.26 |
| Stratified Cox model |  | 0.16 | 0.16 | 0.00 | 95.55 | 0.16 |  | 0.25 | 64.45 | 0.30 |
| Unstratified Cox model without sandwich variance |  | 0.09 | 0.11 | -0.19 | 63.80 | 0.21 |  | 0.06 | 95.75 | 0.11 |
| Unstratified Cox model with sandwich variance |  | 0.09 | 0.09 | -0.19 | 47.35 | 0.21 |  | 0.06 | 90.50 | 0.11 |
|  |  |  |  |  |  |  |  |  |  |  |
| Frailty Cox model | 2 | 0.13 | 0.13 | -0.03 | 94.95 | 0.13 |  | 0.23 | 60.30 | 0.26 |
| Stratified Cox model |  | 0.17 | 0.17 | 0.00 | 95.60 | 0.17 |  | 0.26 | 68.05 | 0.31 |
| Unstratified Cox model without sandwich variance |  | 0.10 | 0.12 | -0.17 | 74.20 | 0.20 |  | 0.08 | 94.55 | 0.13 |
| Unstratified Cox model with sandwich variance |  | 0.10 | 0.10 | -0.17 | 60.05 | 0.20 |  | 0.08 | 88.90 | 0.13 |
|  |  |  |  |  |  |  |  |  |  |  |
| Frailty Cox model | 4 | 0.14 | 0.14 | -0.03 | 94.95 | 0.14 |  | 0.22 | 66.40 | 0.26 |
| Stratified Cox model |  | 0.18 | 0.18 | 0.00 | 95.75 | 0.18 |  | 0.26 | 72.30 | 0.31 |
| Unstratified Cox model without sandwich variance |  | 0.11 | 0.13 | -0.16 | 82.05 | 0.19 |  | 0.10 | 92.95 | 0.15 |
| Unstratified Cox model with sandwich variance |  | 0.11 | 0.11 | -0.16 | 71.60 | 0.19 |  | 0.10 | 87.25 | 0.15 |
|  |  |  |  |  |  |  |  |  |  |  |
| Log conditional-HR = log(1) = 0; Log marginal-HR = 0* | | | | | | | | | | |
| Frailty Cox model | 1 | 0.12 | 0.13 | 0.00 | 96.10 | 0.12 |  | 0.00 | 96.10 | 0.12 |
| Stratified Cox model |  | 0.16 | 0.16 | 0.00 | 95.20 | 0.16 |  | 0.00 | 95.20 | 0.16 |
| Unstratified Cox model without sandwich variance |  | 0.10 | 0.12 | 0.00 | 98.45 | 0.10 |  | 0.00 | 98.45 | 0.10 |
| Unstratified Cox model with sandwich variance |  | 0.10 | 0.10 | 0.00 | 94.80 | 0.10 |  | 0.00 | 94.80 | 0.10 |
|  |  |  |  |  |  |  |  |  |  |  |
| Frailty Cox model | 2 | 0.13 | 0.14 | 0.00 | 96.35 | 0.13 |  | 0.00 | 96.35 | 0.13 |
| Stratified Cox model |  | 0.17 | 0.17 | 0.00 | 95.85 | 0.17 |  | 0.00 | 95.85 | 0.17 |
| Unstratified Cox model without sandwich variance |  | 0.11 | 0.13 | 0.00 | 98.15 | 0.11 |  | 0.00 | 98.15 | 0.11 |
| Unstratified Cox model with sandwich variance |  | 0.11 | 0.11 | 0.00 | 95.35 | 0.11 |  | 0.00 | 95.35 | 0.11 |
|  |  |  |  |  |  |  |  |  |  |  |
| Frailty Cox model | 4 | 0.14 | 0.15 | 0.00 | 95.75 | 0.14 |  | 0.00 | 95.75 | 0.14 |
| Stratified Cox model |  | 0.19 | 0.19 | 0.00 | 95.85 | 0.19 |  | 0.00 | 95.85 | 0.19 |
| Unstratified Cox model without sandwich variance |  | 0.12 | 0.14 | 0.00 | 97.80 | 0.12 |  | 0.00 | 97.80 | 0.12 |
| Unstratified Cox model with sandwich variance |  | 0.12 | 0.12 | 0.00 | 94.75 | 0.12 |  | 0.00 | 94.75 | 0.12 |
|  |  |  |  |  |  |  |  |  |  |  |
| Log conditional-HR = log(0.5) = –0.693; Log marginal-HR = –0.438* | | | | | | | | | | |
| Frailty Cox model | 1 | 0.14 | 0.14 | 0.03 | 95.10 | 0.14 |  | -0.22 | 65.60 | 0.26 |
| Stratified Cox model |  | 0.18 | 0.18 | 0.00 | 95.35 | 0.18 |  | -0.26 | 71.75 | 0.31 |
| Unstratified Cox model without sandwich variance |  | 0.11 | 0.13 | 0.16 | 81.35 | 0.19 |  | -0.10 | 93.35 | 0.15 |
| Unstratified Cox model with sandwich variance |  | 0.11 | 0.11 | 0.16 | 69.95 | 0.19 |  | -0.10 | 86.90 | 0.15 |
|  |  |  |  |  |  |  |  |  |  |  |
| Frailty Cox model | 2 | 0.15 | 0.15 | 0.03 | 95.30 | 0.15 |  | -0.22 | 71.00 | 0.27 |
| Stratified Cox model |  | 0.19 | 0.20 | 0.00 | 95.15 | 0.19 |  | -0.26 | 75.65 | 0.32 |
| Unstratified Cox model without sandwich variance |  | 0.13 | 0.15 | 0.14 | 87.55 | 0.19 |  | -0.12 | 92.55 | 0.17 |
| Unstratified Cox model with sandwich variance |  | 0.13 | 0.13 | 0.14 | 79.60 | 0.19 |  | -0.12 | 87.35 | 0.17 |
|  |  |  |  |  |  |  |  |  |  |  |
| Frailty Cox model | 4 | 0.17 | 0.17 | 0.03 | 95.20 | 0.17 |  | -0.22 | 75.35 | 0.28 |
| Stratified Cox model |  | 0.21 | 0.21 | -0.01 | 95.80 | 0.21 |  | -0.26 | 79.75 | 0.34 |
| Unstratified Cox model without sandwich variance |  | 0.15 | 0.16 | 0.12 | 91.00 | 0.19 |  | -0.14 | 90.75 | 0.20 |
| Unstratified Cox model with sandwich variance |  | 0.15 | 0.15 | 0.12 | 85.85 | 0.19 |  | -0.14 | 85.80 | 0.20 |

HR, hazard ratio; MCSE, empirical (Monte Carlo) standard error; MESE, mean estimated standard error; 95%CP, coverage proportion of 95% confidence interval; RMSE, root mean square error.

*Marginal hazard ratios were calculated under a large (*n* = 5 000 000 pairs) sample in each additional scenario.

Supplementary Table 5. Simulation results from Additional Scenario 1-3: increasing baseline hazard with conditionally independent censoring given matched pairs and exposure (2000 repetitions, *n* = 250).

|  | Censoring rate ratio by exposure | MCSE | MESE | Log conditional-HR | | |  | Log marginal-HR | | |
| --- | --- | --- | --- | --- | --- | --- | --- | --- | --- | --- |
| Method |  |  |  | Bias | 95%CP (%) | RMSE |  | Bias | 95%CP (%) | RMSE |
| Log conditional-HR = log(2) = 0.693; Log marginal-HR = 0.437* | | | | | | | | | | |
| Frailty Cox model | 0.25 | 0.13 | 0.13 | 0.15 | 80.46 | 0.20 |  | 0.40 | 12.63 | 0.43 |
| Stratified Cox model |  | 0.18 | 0.18 | 0.00 | 95.35 | 0.18 |  | 0.25 | 73.20 | 0.31 |
| Unstratified Cox model without sandwich variance |  | 0.10 | 0.12 | 0.00 | 98.15 | 0.10 |  | 0.26 | 39.40 | 0.28 |
| Unstratified Cox model with sandwich variance |  | 0.10 | 0.10 | 0.00 | 95.35 | 0.10 |  | 0.26 | 26.55 | 0.28 |
|  |  |  |  |  |  |  |  |  |  |  |
| Frailty Cox model | 1 | 0.15 | 0.14 | -0.02 | 94.22 | 0.15 |  | 0.24 | 62.19 | 0.28 |
| Stratified Cox model |  | 0.21 | 0.21 | 0.01 | 95.20 | 0.21 |  | 0.26 | 79.15 | 0.34 |
| Unstratified Cox model without sandwich variance |  | 0.11 | 0.13 | -0.15 | 80.15 | 0.19 |  | 0.10 | 91.40 | 0.15 |
| Unstratified Cox model with sandwich variance |  | 0.11 | 0.11 | -0.15 | 70.65 | 0.19 |  | 0.10 | 86.10 | 0.15 |
|  |  |  |  |  |  |  |  |  |  |  |
| Frailty Cox model | 4 | 0.20 | 0.20 | -0.42 | 44.92 | 0.47 |  | -0.16 | 86.27 | 0.26 |
| Stratified Cox model |  | 0.33 | 0.33 | 0.01 | 95.75 | 0.33 |  | 0.26 | 90.40 | 0.42 |
| Unstratified Cox model without sandwich variance |  | 0.17 | 0.18 | -0.54 | 13.80 | 0.57 |  | -0.28 | 67.75 | 0.33 |
| Unstratified Cox model with sandwich variance |  | 0.17 | 0.17 | -0.54 | 11.50 | 0.57 |  | -0.28 | 63.95 | 0.33 |
|  |  |  |  |  |  |  |  |  |  |  |
| Log conditional-HR = log(1) = 0; Log marginal-HR = 0* | | | | | | | | | | |
| Frailty Cox model | 0.25 | 0.13 | 0.13 | 0.20 | 68.17 | 0.24 |  | 0.20 | 68.17 | 0.24 |
| Stratified Cox model |  | 0.18 | 0.18 | 0.00 | 94.85 | 0.18 |  | 0.00 | 94.85 | 0.18 |
| Unstratified Cox model without sandwich variance |  | 0.10 | 0.12 | 0.22 | 57.80 | 0.24 |  | 0.22 | 57.80 | 0.24 |
| Unstratified Cox model with sandwich variance |  | 0.10 | 0.10 | 0.22 | 44.50 | 0.24 |  | 0.22 | 44.50 | 0.24 |
|  |  |  |  |  |  |  |  |  |  |  |
| Frailty Cox model | 1 | 0.14 | 0.15 | 0.00 | 95.57 | 0.14 |  | 0.00 | 95.57 | 0.14 |
| Stratified Cox model |  | 0.22 | 0.21 | 0.00 | 95.10 | 0.22 |  | 0.00 | 95.10 | 0.22 |
| Unstratified Cox model without sandwich variance |  | 0.12 | 0.13 | 0.00 | 97.70 | 0.12 |  | 0.00 | 97.70 | 0.12 |
| Unstratified Cox model with sandwich variance |  | 0.12 | 0.12 | 0.00 | 95.40 | 0.12 |  | 0.00 | 95.40 | 0.12 |
|  |  |  |  |  |  |  |  |  |  |  |
| Frailty Cox model | 4 | 0.23 | 0.23 | -0.44 | 51.92 | 0.49 |  | -0.44 | 51.92 | 0.49 |
| Stratified Cox model |  | 0.35 | 0.35 | -0.01 | 96.35 | 0.35 |  | -0.01 | 96.35 | 0.35 |
| Unstratified Cox model without sandwich variance |  | 0.20 | 0.21 | -0.47 | 39.35 | 0.52 |  | -0.47 | 39.35 | 0.52 |
| Unstratified Cox model with sandwich variance |  | 0.20 | 0.20 | -0.47 | 34.65 | 0.52 |  | -0.47 | 34.65 | 0.52 |
|  |  |  |  |  |  |  |  |  |  |  |
| Log conditional-HR = log(0.5) = –0.693; Log marginal-HR = –0.438* | | | | | | | | | | |
| Frailty Cox model | 0.25 | 0.14 | 0.14 | 0.24 | 56.27 | 0.28 |  | -0.01 | 94.48 | 0.14 |
| Stratified Cox model |  | 0.20 | 0.20 | -0.01 | 95.60 | 0.20 |  | -0.26 | 76.75 | 0.33 |
| Unstratified Cox model without sandwich variance |  | 0.11 | 0.12 | 0.41 | 5.95 | 0.42 |  | 0.16 | 78.00 | 0.19 |
| Unstratified Cox model with sandwich variance |  | 0.11 | 0.11 | 0.41 | 3.80 | 0.42 |  | 0.16 | 70.20 | 0.19 |
|  |  |  |  |  |  |  |  |  |  |  |
| Frailty Cox model | 1 | 0.16 | 0.16 | 0.02 | 95.55 | 0.16 |  | -0.24 | 69.80 | 0.29 |
| Stratified Cox model |  | 0.24 | 0.24 | -0.01 | 95.65 | 0.24 |  | -0.26 | 82.80 | 0.36 |
| Unstratified Cox model without sandwich variance |  | 0.13 | 0.15 | 0.13 | 87.35 | 0.19 |  | -0.12 | 89.95 | 0.18 |
| Unstratified Cox model with sandwich variance |  | 0.13 | 0.13 | 0.13 | 81.80 | 0.19 |  | -0.12 | 85.85 | 0.18 |
|  |  |  |  |  |  |  |  |  |  |  |
| Frailty Cox model | 4 | 0.28 | 0.28 | -0.46 | 65.54 | 0.53 |  | -0.71 | 24.73 | 0.76 |
| Stratified Cox model |  | 0.42 | 0.42 | -0.03 | 96.30 | 0.42 |  | -0.29 | 93.45 | 0.51 |
| Unstratified Cox model without sandwich variance |  | 0.26 | 0.27 | -0.43 | 68.25 | 0.50 |  | -0.68 | 23.55 | 0.73 |
| Unstratified Cox model with sandwich variance |  | 0.26 | 0.25 | -0.43 | 63.20 | 0.50 |  | -0.68 | 19.75 | 0.73 |

HR, hazard ratio; MCSE, empirical (Monte Carlo) standard error; MESE, mean estimated standard error; 95%CP, coverage proportion of 95% confidence interval; RMSE, root mean square error.

*Marginal hazard ratios were calculated under a large (*n* = 5 000 000 pairs) sample in each additional scenario.

Supplementary Table 6. Simulation results from Additional Scenario 1-4: decreasing baseline hazard with conditionally independent censoring given matched pairs and exposure (2000 repetitions, *n* = 250).

|  | Censoring rate ratio by exposure | MCSE | MESE | Log conditional-HR | | |  | Log marginal-HR | | |
| --- | --- | --- | --- | --- | --- | --- | --- | --- | --- | --- |
| Method |  |  |  | Bias | 95%CP (%) | RMSE |  | Bias | 95%CP (%) | RMSE |
| Log conditional-HR = log(2) = 0.693; Log marginal-HR = 0.437* | | | | | | | | | | |
| Frailty Cox model | 0.25 | 0.12 | 0.12 | 0.04 | 93.49 | 0.13 |  | 0.30 | 28.83 | 0.32 |
| Stratified Cox model |  | 0.15 | 0.15 | 0.00 | 94.20 | 0.15 |  | 0.26 | 60.30 | 0.30 |
| Unstratified Cox model without sandwich variance |  | 0.09 | 0.11 | -0.10 | 89.35 | 0.14 |  | 0.15 | 76.40 | 0.17 |
| Unstratified Cox model with sandwich variance |  | 0.09 | 0.09 | -0.10 | 78.75 | 0.14 |  | 0.15 | 61.85 | 0.17 |
|  |  |  |  |  |  |  |  |  |  |  |
| Frailty Cox model | 1 | 0.12 | 0.12 | -0.02 | 94.65 | 0.12 |  | 0.24 | 51.08 | 0.27 |
| Stratified Cox model |  | 0.16 | 0.15 | 0.00 | 94.90 | 0.16 |  | 0.26 | 62.45 | 0.30 |
| Unstratified Cox model without sandwich variance |  | 0.09 | 0.11 | -0.16 | 73.50 | 0.19 |  | 0.09 | 92.80 | 0.13 |
| Unstratified Cox model with sandwich variance |  | 0.09 | 0.09 | -0.16 | 58.50 | 0.19 |  | 0.09 | 83.70 | 0.13 |
|  |  |  |  |  |  |  |  |  |  |  |
| Frailty Cox model | 4 | 0.13 | 0.13 | -0.12 | 84.24 | 0.18 |  | 0.13 | 82.29 | 0.19 |
| Stratified Cox model |  | 0.17 | 0.17 | 0.00 | 94.65 | 0.17 |  | 0.26 | 68.90 | 0.31 |
| Unstratified Cox model without sandwich variance |  | 0.11 | 0.12 | -0.26 | 42.25 | 0.28 |  | -0.01 | 97.55 | 0.11 |
| Unstratified Cox model with sandwich variance |  | 0.11 | 0.11 | -0.26 | 30.25 | 0.28 |  | -0.01 | 94.60 | 0.11 |
|  |  |  |  |  |  |  |  |  |  |  |
| Log conditional-HR = log(1) = 0; Log marginal-HR = 0* | | | | | | | | | | |
| Frailty Cox model | 0.25 | 0.12 | 0.12 | 0.08 | 91.35 | 0.14 |  | 0.08 | 91.35 | 0.14 |
| Stratified Cox model |  | 0.15 | 0.15 | 0.00 | 95.45 | 0.15 |  | 0.00 | 95.45 | 0.15 |
| Unstratified Cox model without sandwich variance |  | 0.09 | 0.11 | 0.10 | 91.40 | 0.14 |  | 0.10 | 91.40 | 0.14 |
| Unstratified Cox model with sandwich variance |  | 0.09 | 0.10 | 0.10 | 81.75 | 0.14 |  | 0.10 | 81.75 | 0.14 |
|  |  |  |  |  |  |  |  |  |  |  |
| Frailty Cox model | 1 | 0.12 | 0.13 | 0.00 | 95.35 | 0.12 |  | 0.00 | 95.35 | 0.12 |
| Stratified Cox model |  | 0.16 | 0.16 | 0.00 | 95.30 | 0.16 |  | 0.00 | 95.30 | 0.16 |
| Unstratified Cox model without sandwich variance |  | 0.10 | 0.12 | 0.00 | 97.55 | 0.10 |  | 0.00 | 97.55 | 0.10 |
| Unstratified Cox model with sandwich variance |  | 0.10 | 0.10 | 0.00 | 95.00 | 0.10 |  | 0.00 | 95.00 | 0.10 |
|  |  |  |  |  |  |  |  |  |  |  |
| Frailty Cox model | 4 | 0.14 | 0.14 | -0.12 | 87.54 | 0.19 |  | -0.12 | 87.54 | 0.19 |
| Stratified Cox model |  | 0.18 | 0.18 | 0.00 | 94.85 | 0.18 |  | 0.00 | 94.85 | 0.18 |
| Unstratified Cox model without sandwich variance |  | 0.12 | 0.14 | -0.15 | 85.10 | 0.19 |  | -0.15 | 85.10 | 0.19 |
| Unstratified Cox model with sandwich variance |  | 0.12 | 0.12 | -0.15 | 77.10 | 0.19 |  | -0.15 | 77.10 | 0.19 |
|  |  |  |  |  |  |  |  |  |  |  |
| Log conditional-HR = log(0.5) = –0.693; Log marginal-HR = –0.438* | | | | | | | | | | |
| Frailty Cox model | 0.25 | 0.13 | 0.13 | 0.12 | 85.05 | 0.18 |  | -0.13 | 82.65 | 0.19 |
| Stratified Cox model |  | 0.17 | 0.17 | 0.00 | 95.70 | 0.17 |  | -0.26 | 69.70 | 0.31 |
| Unstratified Cox model without sandwich variance |  | 0.11 | 0.12 | 0.26 | 41.65 | 0.29 |  | 0.01 | 97.20 | 0.11 |
| Unstratified Cox model with sandwich variance |  | 0.11 | 0.11 | 0.26 | 29.10 | 0.29 |  | 0.01 | 94.90 | 0.11 |
|  |  |  |  |  |  |  |  |  |  |  |
| Frailty Cox model | 1 | 0.14 | 0.14 | 0.02 | 95.29 | 0.14 |  | -0.23 | 64.43 | 0.27 |
| Stratified Cox model |  | 0.18 | 0.18 | 0.00 | 95.00 | 0.18 |  | -0.26 | 72.55 | 0.31 |
| Unstratified Cox model without sandwich variance |  | 0.12 | 0.14 | 0.12 | 89.15 | 0.17 |  | -0.14 | 86.50 | 0.18 |
| Unstratified Cox model with sandwich variance |  | 0.12 | 0.12 | 0.12 | 83.35 | 0.17 |  | -0.14 | 78.90 | 0.18 |
|  |  |  |  |  |  |  |  |  |  |  |
| Frailty Cox model | 4 | 0.16 | 0.17 | -0.12 | 90.63 | 0.20 |  | -0.38 | 37.64 | 0.41 |
| Stratified Cox model |  | 0.20 | 0.21 | -0.01 | 95.05 | 0.20 |  | -0.26 | 78.55 | 0.33 |
| Unstratified Cox model without sandwich variance |  | 0.15 | 0.17 | -0.08 | 95.60 | 0.17 |  | -0.34 | 46.85 | 0.37 |
| Unstratified Cox model with sandwich variance |  | 0.15 | 0.15 | -0.08 | 92.60 | 0.17 |  | -0.34 | 38.05 | 0.37 |

HR, hazard ratio; MCSE, empirical (Monte Carlo) standard error; MESE, mean estimated standard error; 95%CP, coverage proportion of 95% confidence interval; RMSE, root mean square error.

*Marginal hazard ratios were calculated under a large (*n* = 5 000 000 pairs) sample in each additional scenario.

Supplementary Table 7. Simulation results from additional scenario 2-1: varying a Weibull shape parameter across pairs, with independent censoring (2,000 Repetitions, *n* = 250).

|  | Censoring rate | MCSE | MESE | Log conditional-HR | | |  | Log marginal-HR | | |
| --- | --- | --- | --- | --- | --- | --- | --- | --- | --- | --- |
| Method |  |  |  | Bias | 95%CP (%) | RMSE |  | Bias | 95%CP (%) | RMSE |
| Log conditional-HR = log(2) = 0.693; Log marginal-HR = 0.411* | | | | | | | | | | |
| Frailty Cox model | 1 | 0.13 | 0.13 | -0.03 | 94.35 | 0.14 |  | 0.25 | 54.73 | 0.28 |
| Stratified Cox model |  | 0.18 | 0.18 | 0.01 | 95.30 | 0.18 |  | 0.29 | 64.40 | 0.34 |
| Unstratified Cox model without sandwich variance |  | 0.10 | 0.12 | -0.21 | 60.30 | 0.23 |  | 0.07 | 95.00 | 0.12 |
| Unstratified Cox model with sandwich variance |  | 0.10 | 0.10 | -0.21 | 45.20 | 0.23 |  | 0.07 | 89.10 | 0.12 |
|  |  |  |  |  |  |  |  |  |  |  |
| Frailty Cox model | 2 | 0.15 | 0.15 | -0.03 | 94.45 | 0.15 |  | 0.25 | 63.45 | 0.29 |
| Stratified Cox model |  | 0.20 | 0.20 | 0.01 | 95.55 | 0.20 |  | 0.29 | 72.55 | 0.35 |
| Unstratified Cox model without sandwich variance |  | 0.12 | 0.14 | -0.19 | 76.40 | 0.22 |  | 0.10 | 94.05 | 0.15 |
| Unstratified Cox model with sandwich variance |  | 0.12 | 0.12 | -0.19 | 64.45 | 0.22 |  | 0.10 | 88.05 | 0.15 |
|  |  |  |  |  |  |  |  |  |  |  |
| Frailty Cox model | 4 | 0.17 | 0.18 | -0.04 | 95.15 | 0.18 |  | 0.24 | 74.85 | 0.29 |
| Stratified Cox model |  | 0.24 | 0.24 | 0.01 | 95.50 | 0.24 |  | 0.29 | 78.75 | 0.38 |
| Unstratified Cox model without sandwich variance |  | 0.15 | 0.17 | -0.17 | 86.00 | 0.22 |  | 0.11 | 93.35 | 0.18 |
| Unstratified Cox model with sandwich variance |  | 0.15 | 0.14 | -0.17 | 76.50 | 0.22 |  | 0.11 | 88.65 | 0.18 |
|  |  |  |  |  |  |  |  |  |  |  |
| Log conditional-HR = log(1) = 0; Log marginal-HR = 0* | | | | | | | | | | |
| Frailty Cox model | 1 | 0.13 | 0.14 | 0.00 | 96.35 | 0.13 |  | 0.00 | 96.35 | 0.13 |
| Stratified Cox model |  | 0.18 | 0.18 | 0.00 | 94.70 | 0.18 |  | 0.00 | 94.70 | 0.18 |
| Unstratified Cox model without sandwich variance |  | 0.11 | 0.13 | 0.00 | 98.40 | 0.11 |  | 0.00 | 98.40 | 0.11 |
| Unstratified Cox model with sandwich variance |  | 0.11 | 0.11 | 0.00 | 94.90 | 0.11 |  | 0.00 | 94.90 | 0.11 |
|  |  |  |  |  |  |  |  |  |  |  |
| Frailty Cox model | 2 | 0.16 | 0.16 | 0.00 | 96.40 | 0.16 |  | 0.00 | 96.40 | 0.16 |
| Stratified Cox model |  | 0.22 | 0.21 | 0.00 | 95.10 | 0.22 |  | 0.00 | 95.10 | 0.22 |
| Unstratified Cox model without sandwich variance |  | 0.13 | 0.15 | 0.00 | 97.90 | 0.13 |  | 0.00 | 97.90 | 0.13 |
| Unstratified Cox model with sandwich variance |  | 0.13 | 0.13 | 0.00 | 95.75 | 0.13 |  | 0.00 | 95.75 | 0.13 |
|  |  |  |  |  |  |  |  |  |  |  |
| Frailty Cox model | 4 | 0.19 | 0.19 | 0.00 | 96.30 | 0.19 |  | 0.00 | 96.30 | 0.19 |
| Stratified Cox model |  | 0.27 | 0.25 | 0.00 | 94.95 | 0.27 |  | 0.00 | 94.95 | 0.27 |
| Unstratified Cox model without sandwich variance |  | 0.16 | 0.18 | 0.00 | 97.85 | 0.16 |  | 0.00 | 97.85 | 0.16 |
| Unstratified Cox model with sandwich variance |  | 0.16 | 0.16 | 0.00 | 94.90 | 0.16 |  | 0.00 | 94.90 | 0.16 |
|  |  |  |  |  |  |  |  |  |  |  |
| Log conditional-HR = log(0.5) = –0.693; Log marginal-HR = –0.398* | | | | | | | | | | |
| Frailty Cox model | 1 | 0.15 | 0.15 | 0.05 | 94.80 | 0.16 |  | -0.25 | 62.18 | 0.29 |
| Stratified Cox model |  | 0.21 | 0.20 | -0.01 | 94.80 | 0.21 |  | -0.30 | 67.95 | 0.37 |
| Unstratified Cox model without sandwich variance |  | 0.12 | 0.14 | 0.18 | 76.45 | 0.22 |  | -0.11 | 91.70 | 0.16 |
| Unstratified Cox model with sandwich variance |  | 0.12 | 0.12 | 0.18 | 66.20 | 0.22 |  | -0.11 | 86.00 | 0.16 |
|  |  |  |  |  |  |  |  |  |  |  |
| Frailty Cox model | 2 | 0.18 | 0.18 | 0.04 | 94.79 | 0.18 |  | -0.25 | 70.97 | 0.31 |
| Stratified Cox model |  | 0.26 | 0.24 | -0.01 | 94.90 | 0.26 |  | -0.31 | 75.55 | 0.40 |
| Unstratified Cox model without sandwich variance |  | 0.15 | 0.17 | 0.15 | 87.70 | 0.21 |  | -0.15 | 90.50 | 0.21 |
| Unstratified Cox model with sandwich variance |  | 0.15 | 0.15 | 0.15 | 81.05 | 0.21 |  | -0.15 | 84.30 | 0.21 |
|  |  |  |  |  |  |  |  |  |  |  |
| Frailty Cox model | 4 | 0.21 | 0.22 | 0.03 | 95.83 | 0.22 |  | -0.26 | 79.42 | 0.34 |
| Stratified Cox model |  | 0.31 | 0.30 | -0.03 | 94.90 | 0.31 |  | -0.32 | 82.70 | 0.45 |
| Unstratified Cox model without sandwich variance |  | 0.19 | 0.21 | 0.12 | 93.20 | 0.22 |  | -0.18 | 90.45 | 0.26 |
| Unstratified Cox model with sandwich variance |  | 0.19 | 0.19 | 0.12 | 89.65 | 0.22 |  | -0.18 | 85.15 | 0.26 |

HR, hazard ratio; MCSE, empirical (Monte Carlo) standard error; MESE, mean estimated standard error; 95%CP, coverage proportion of 95% confidence interval; RMSE, root mean square error.

*Marginal hazard ratios were calculated under a large (*n* = 5 000 000 pairs) sample in each additional scenario.

Supplementary Table 8. Simulation results from additional scenario 2-2: varying a Weibull shape parameter across pairs, with conditionally independent censoring given matched pairs and exposure (2,000 Repetitions, *n* = 250).

|  | Censoring rate ratio by exposure | MCSE | MESE | Log conditional-HR | | |  | Log marginal-HR | | |
| --- | --- | --- | --- | --- | --- | --- | --- | --- | --- | --- |
| Method |  |  |  | Bias | 95%CP (%) | RMSE |  | Bias | 95%CP (%) | RMSE |
| Log conditional-HR = log(2) = 0.693; Log marginal-HR = 0.411* | | | | | | | | | | |
| Frailty Cox model | 0.25 | 0.13 | 0.12 | 0.07 | 90.40 | 0.15 |  | 0.36 | 17.05 | 0.38 |
| Stratified Cox model |  | 0.16 | 0.16 | 0.00 | 95.85 | 0.16 |  | 0.28 | 59.55 | 0.33 |
| Unstratified Cox model without sandwich variance |  | 0.09 | 0.11 | -0.08 | 92.55 | 0.12 |  | 0.20 | 60.00 | 0.22 |
| Unstratified Cox model with sandwich variance |  | 0.09 | 0.09 | -0.08 | 86.50 | 0.12 |  | 0.20 | 44.15 | 0.22 |
|  |  |  |  |  |  |  |  |  |  |  |
| Frailty Cox model | 1 | 0.13 | 0.13 | -0.03 | 94.14 | 0.13 |  | 0.25 | 52.85 | 0.28 |
| Stratified Cox model |  | 0.18 | 0.18 | 0.00 | 95.60 | 0.18 |  | 0.28 | 65.30 | 0.33 |
| Unstratified Cox model without sandwich variance |  | 0.10 | 0.12 | -0.18 | 68.90 | 0.21 |  | 0.10 | 90.70 | 0.14 |
| Unstratified Cox model with sandwich variance |  | 0.10 | 0.10 | -0.18 | 55.85 | 0.21 |  | 0.10 | 84.00 | 0.14 |
|  |  |  |  |  |  |  |  |  |  |  |
| Frailty Cox model | 4 | 0.15 | 0.16 | -0.25 | 65.53 | 0.29 |  | 0.04 | 94.49 | 0.16 |
| Stratified Cox model |  | 0.21 | 0.22 | 0.00 | 96.00 | 0.21 |  | 0.28 | 76.05 | 0.35 |
| Unstratified Cox model without sandwich variance |  | 0.13 | 0.15 | -0.39 | 20.30 | 0.41 |  | -0.11 | 91.35 | 0.17 |
| Unstratified Cox model with sandwich variance |  | 0.13 | 0.13 | -0.39 | 14.80 | 0.41 |  | -0.11 | 87.20 | 0.17 |
|  |  |  |  |  |  |  |  |  |  |  |
| Log conditional-HR = log(1) = 0; Log marginal-HR = 0* | | | | | | | | | | |
| Frailty Cox model | 0.25 | 0.12 | 0.13 | 0.13 | 82.88 | 0.18 |  | 0.13 | 82.88 | 0.18 |
| Stratified Cox model |  | 0.16 | 0.16 | 0.00 | 95.45 | 0.16 |  | 0.00 | 95.45 | 0.16 |
| Unstratified Cox model without sandwich variance |  | 0.10 | 0.12 | 0.15 | 78.45 | 0.18 |  | 0.15 | 78.45 | 0.18 |
| Unstratified Cox model with sandwich variance |  | 0.10 | 0.10 | 0.15 | 66.15 | 0.18 |  | 0.15 | 66.15 | 0.18 |
|  |  |  |  |  |  |  |  |  |  |  |
| Frailty Cox model | 1 | 0.13 | 0.14 | 0.00 | 95.60 | 0.13 |  | 0.00 | 95.60 | 0.13 |
| Stratified Cox model |  | 0.18 | 0.18 | 0.00 | 95.80 | 0.18 |  | 0.00 | 95.80 | 0.18 |
| Unstratified Cox model without sandwich variance |  | 0.11 | 0.13 | 0.00 | 98.10 | 0.11 |  | 0.00 | 98.10 | 0.11 |
| Unstratified Cox model with sandwich variance |  | 0.11 | 0.11 | 0.00 | 95.00 | 0.11 |  | 0.00 | 95.00 | 0.11 |
|  |  |  |  |  |  |  |  |  |  |  |
| Frailty Cox model | 4 | 0.17 | 0.18 | -0.24 | 74.55 | 0.29 |  | -0.24 | 74.55 | 0.29 |
| Stratified Cox model |  | 0.23 | 0.23 | 0.00 | 95.45 | 0.23 |  | 0.00 | 95.45 | 0.23 |
| Unstratified Cox model without sandwich variance |  | 0.15 | 0.17 | -0.28 | 64.00 | 0.31 |  | -0.28 | 64.00 | 0.31 |
| Unstratified Cox model with sandwich variance |  | 0.15 | 0.15 | -0.28 | 56.00 | 0.31 |  | -0.28 | 56.00 | 0.31 |
|  |  |  |  |  |  |  |  |  |  |  |
| Log conditional-HR = log(0.5) = –0.693; Log marginal-HR = –0.398* | | | | | | | | | | |
| Frailty Cox model | 0.25 | 0.13 | 0.13 | 0.21 | 66.05 | 0.24 |  | -0.09 | 90.09 | 0.16 |
| Stratified Cox model |  | 0.18 | 0.18 | -0.01 | 94.90 | 0.18 |  | -0.30 | 63.25 | 0.35 |
| Unstratified Cox model without sandwich variance |  | 0.11 | 0.12 | 0.36 | 13.65 | 0.37 |  | 0.06 | 95.35 | 0.12 |
| Unstratified Cox model with sandwich variance |  | 0.11 | 0.11 | 0.36 | 8.60 | 0.37 |  | 0.06 | 90.80 | 0.12 |
|  |  |  |  |  |  |  |  |  |  |  |
| Frailty Cox model | 1 | 0.15 | 0.15 | 0.04 | 94.73 | 0.15 |  | -0.26 | 60.77 | 0.30 |
| Stratified Cox model |  | 0.21 | 0.21 | -0.01 | 94.65 | 0.21 |  | -0.30 | 69.70 | 0.37 |
| Unstratified Cox model without sandwich variance |  | 0.12 | 0.14 | 0.14 | 84.45 | 0.19 |  | -0.15 | 84.45 | 0.20 |
| Unstratified Cox model with sandwich variance |  | 0.12 | 0.13 | 0.14 | 78.10 | 0.19 |  | -0.15 | 78.45 | 0.20 |
|  |  |  |  |  |  |  |  |  |  |  |
| Frailty Cox model | 4 | 0.21 | 0.21 | -0.23 | 83.22 | 0.31 |  | -0.53 | 28.96 | 0.57 |
| Stratified Cox model |  | 0.28 | 0.27 | -0.02 | 95.10 | 0.28 |  | -0.31 | 80.45 | 0.42 |
| Unstratified Cox model without sandwich variance |  | 0.19 | 0.21 | -0.21 | 86.50 | 0.28 |  | -0.50 | 28.20 | 0.54 |
| Unstratified Cox model with sandwich variance |  | 0.19 | 0.19 | -0.21 | 83.30 | 0.28 |  | -0.50 | 22.75 | 0.54 |

HR, hazard ratio; MCSE, empirical (Monte Carlo) standard error; MESE, mean estimated standard error; 95%CP, coverage proportion of 95% confidence interval; RMSE, root mean square error.

*Marginal hazard ratios were calculated under a large (*n* = 5 000 000 pairs) sample in each additional scenario.

1. **SAS program for matching women in the Rotterdam tumor database based on estimated propensity scores**

/*--------------------------------------

Variables in the Rotterdam dataset (rott2)

-Time: RF

-Event: RFI (0/1)

-Exposure: NO_CHEMO (0/1)

-Covariates:

AGE

MENO (characteristic 0/1)

SIZE (characteristic 3-category)

GRADE (characteristic 2/3)

NODES (0-34; transformed into exp(-0.12*NODES))

PR

ER

-Patient ID: PID

-----------------------------------------*/

/*---------- Estimating PS ----------*/

**data** rott2;

set rott2;

ex_NODES = exp(-**0.12***NODES);

**run**;

**proc** **logistic** data = rott2 desc;

class MENO SIZE GRADE;

model NO_CHEMO = AGE MENO SIZE GRADE ex_NODES PR ER;

output out = rott2 p = PS;

**run**;

**data** rott2;

set rott2;

CHEMO = (**1** - NO_CHEMO);

W = NO_CHEMO/PS + (**1** - NO_CHEMO)/(**1** - PS);

**run**;

/*---------- Matching on PS ----------*/

**proc** **means** std data = rott2;

var PS;

where NO_CHEMO = **0**;

output out = PS_std std = PS_std;

**run**;

* SD(PS|Chemotherapy) = 0.23;

**data** PS_std;

set PS_std;

call symput("std_PS", **0.2** * PS_std);

**run**;

%include 'C:\gmatch.sas';

/* Macro is available from Mayo Clinic website

http://www.mayo.edu/research/departments-divisions/department-health-sciences-research/division-biomedical-statistics-informatics/software/locally-written-sas-macros */

%***gmatch***(

data = rott2,

group = CHEMO,

id = PID,

mvars = PS,

wts = **1**,

dist = **1**,

dmaxk = &std_PS,

ncontls = **1**,

seedca = **123**,

seedco = **123**,

out = rott_m,

print = N

);

**data** rott_m1;

set rott_m;

PID = __IDCA;

PAIR = _n_;

keep pid pair;

**data** rott_m0;

set rott_m;

PID = __IDCO;

PAIR = _n_;

keep PID PAIR;

**proc** **sort** data = rott2;

by PID;

**proc** **sort** data = rott_m1;

by PID;

**proc** **sort** data = rott_m0;

by PID;

**run**;

**data** rott3;

merge rott2 rott_m1 rott_m0;

by PID;

if PAIR = **.** then delete;

**run**;

* Matched set: rott3;

* Original set: rott2;

/*---------- Analysis by Cox models ----------*/

* Conditional HR (matched set);

**proc** **phreg** data = rott3;

model RF * RFI(**0**) = NO_CHEMO/ rl ties=exact;

strata PAIR;

**run**;

* Marginal HR (matched set);

**proc** **phreg** data = rott3 covs(aggregate);

model RF * RFI(**0**) = NO_CHEMO/ rl ties=exact;

id PAIR;

**run**;

* IPW (original set);

**proc** **phreg** data = rott2 covs(aggregate);

model RF * RFI(**0**) = NO_CHEMO/ rl;

weight W;

id PID;

**run**;

* Multivariable-adjusted (original set);

**proc** **phreg** data = rott2;

class MENO SIZE GRADE;

model RF * RFI(**0**) = NO_CHEMO AGE MENO SIZE GRADE ex_NODES PR ER AGE*AGE ex_NODES*ex_NODES / rl;

**run**;

* IPW + Multivariable-adjusted (original set);

**proc** **phreg** data = rott2 covs(aggregate);

class MENO SIZE GRADE;

model RF * RFI(**0**) = NO_CHEMO AGE MENO SIZE GRADE ex_NODES PR ER AGE*AGE ex_NODES*ex_NODES / rl;

weight W;

id PID;

**run**;

* Unadjusted (original set);

**proc** **phreg** data = rott2;

model RF * RFI(**0**) = NO_CHEMO/ rl;

**run**;
